# Therapeutic alliance in individual adult psychotherapy: a systematic review of conceptualizations and measures for face-to-face- and online-psychotherapy

**DOI:** 10.3389/fpsyg.2024.1293851

**Published:** 2024-06-27

**Authors:** Eva Saxler, Theresa Schindler, Alexandra Philipsen, Marcel Schulze, Silke Lux

**Affiliations:** ^1^Deutsches Zentrum für Neurodegnerative Erkrankungen, University Clinic of Bonn, Bonn, Germany; ^2^Department of Psychiatry and Psychotherapy, University Hospital Bonn, Bonn, Germany

**Keywords:** therapeutic alliance, measurements, conceptualizations, psychotherapy, systematic review

## Abstract

**Background:**

The therapeutic alliance (TA) is a robust and pantheoretical predictor of treatment outcome in Face-to-Face- (F2F-) and Online-psychotherapy (Online-PT). Many authors have proposed several conceptualizations of TA, which are oftentimes operationalized. The resulting diversity of conceptualizations and measures is presented in this review.

**Methods:**

We performed a three-parted literature search for self-report-instruments of TA in individual, voluntary F2F-PT with adults (1. utilization of past reviews, 2. systematic literature search yielding 5,205 articles, 3. reference lists). Analogously, we conducted a systematic literature search for instruments of TA in the Online-setting (yielding 200 articles). Additionally, we analyzed the content of the instruments qualitatively.

**Results:**

A current overview of 48 instruments for measuring TA (46 for F2F-PT, 2 for Online-PT) including their conceptual backgrounds, characteristics and main content aspects is presented. Most instruments (*n* = 24) operationalize one or more theoretical conceptualizations of TA. Other instruments are adaptation/syntheses of existing measures (*n* = 14), based on literature searches (*n* = 3) or on an empirical survey (*n* = 3) and two instruments provide no conceptual background information. The content of the instruments mainly focused on the following aspects: 1. Self-disclosure and authenticity; 2. Agreement; 3. Active participation, motivation and compliance; 4. Trust and secure attachment; and 5. Considering needs/abilities/wishes of the patient. Additionally, a narrative review of various approaches to conceptualize TA is presented and linked to respective corresponding instruments.

**Discussion:**

The broad variety of conceptualizations and measures of TA makes coherent research on TA difficult. There are conceptual challenges such as the role of attachment style in TA that remain to be clarified. The current conceptualizations and measures do not incorporate the practical experience and expertise of psychotherapists and patients sufficiently. A metatheoretical conceptualization and measure of TA based on an empirical survey of psychotherapists and patients could address these issues.

## 1 Introduction

The therapeutic alliance (TA) is regarded as a robust predictor of psychotherapy (PT) success (Martin et al., 2000; Horvath et al., 2011; Flückiger et al., 2012, 2018, 2020; Del Re et al., 2021). The correlation between TA and PT outcome (“alliance-outcome-correlation”) is reported around *r* = 0.28 and remains significant throughout different types of psychotherapy, so that it is considered a pantheoretical factor (Flückiger et al., 2012, 2018; Del Re et al., 2021). Even though TA has been of central interest in PT-research for years, there are gaps in the conceptualization and measurement of this factor (Elvins and Green, 2008; Del Re et al., 2021). Over the last century, many authors have presented different theoretical conceptualizations of TA and many corresponding questionnaires have been developed (Elvins and Green, 2008; Ardito and Rabellino, 2011; Flückiger et al., 2018; Horvath, 2018). Elvins and Green already spoke of “significant deficiencies” concerning the conceptualization and measurement of TA in 2008 (p. 1168). The authors further identified 35 different instruments for measuring TA. The development continues and the number of instruments is expected to keep increasing (Horvath, 2018). In their recent article, Del Re et al. (2021) come to the conclusion that is still unresolved, which therapist-related traits, skills or behaviors are helpful for the establishment of a good TA. The large variety of theoretical conceptualizations and respective measurements makes it difficult to do research on a single and consistent construct.

One conceptual imprecision that seems particularly important concerns the distinction of TA from the patient’s attachment style to the therapist (e.g., Strauß and Schwark, 2007; Obegi, 2008; Lilliengren et al., 2015; Mallinckrodt and Jeong, 2015; Mallinckrodt et al., 2017; Kim, 2018; Petrowski et al., 2019). It seems clear that the general attachment style of patients, which already exists before starting psychotherapy, seems to have an impact on TA (e.g., Strauß and Schwark, 2007; Mallinckrodt and Jeong, 2015; Mallinckrodt et al., 2017). However, the conceptual and empirical relation between TA and the patient’s particular attachment style to the therapist still remains unclear.

Furthermore, the clarification of role, conceptualization and measurement of TA in Online-PT is a relatively new challenge. TA seems to remain a significant predictor of treatment success in Online-PT (Flückiger et al., 2018; Kaiser et al., 2021). However, reported alliance-outcome-correlations for Face-to-Face- (F2F) and Online-PT are sometimes nearly identical (respective *r* = 0.278 vs. *r* = 0.275; Flückiger et al., 2018), and sometimes the alliance-outcome-correlation seems to be lower in Online-PT (*r* = 0.20; Kaiser et al., 2021). Some authors question the comparability of TA between both forms of PT altogether (Gómez Penedo et al., 2020).

The first goal of this article is to provide a current review of measurements of TA in F2F-PT, as well as in Online-PT along with their conceptual backgrounds and characteristics. The items of the retrieved instruments shall be analyzed so that the main content aspects of TA in current instruments can be presented clearly. The second goal is to provide detailed information about the according conceptualizations of TA and their interdependent origins in a narrative form. One important question hereby will be to which extent not only theoretical, but also empirical approaches toward defining TA will be reflected by current conceptualizations and measurements of TA.

## 2 Methods

### 2.1 Systematic literature search

In order to provide an overview of measurements, we performed two separate literature searches for measurements of TA in F2F- and in Online-PT. The inclusion criteria for the articles were formulated based on the COnsensus-based Standards for the selection of health Measurement Instruments (COSMIN-guidelines) (Prinsen et al., 2018). These describe a systematic approach to reporting via self-report questionnaires [Patient-Reported Outcome Measures (PROMs)]. According to the COSMIN guidelines, the construct to be measured, the population of interest, the type of instrument, and relevant psychometric properties of the instrument should be defined in the inclusion criteria. The last criterion (psychometric properties) was not specified, as the aim was to identify as many current measurement instruments as possible. Accordingly, we did not analyze the quality of the studies, as we were interested in including every instrument that could in theory be used by researchers and clinicians to measure TA in the setting clarified below. The analysis steps of the selection process were based on the Preferred Reporting Items for Systematic review and Meta-Analysis protocols (PRISMA) (Shamseer et al., 2015; Page et al., 2021). This review was not pre-registered.

#### 2.1.1 Therapeutic alliance in face-to-face-psychotherapy

In order to obtain an extensive list of measurements of the TA, we drew upon two existing systematic reviews (Elvins and Green, 2008; Ardito and Rabellino, 2011), conducted a systematic literature search ourselves and identified further articles in reference lists. The inclusion criteria for articles were:

- Construct: The article should present at least one instrument for measuring TA (e.g., development, validation or review of the instrument).
- Population: The presented instrument should be applicable in voluntary individual F2F-PT with adult patients provided by psychologists or psychiatrists.
- Instrument type: The presented instrument should be a self-report or an observer-based measure of TA for patients, therapists or observers.
- Psychometric properties: These were not further specified, because our goal was to present as many existing instruments as possible. However, a complete copy of the items should be included in the cited source if possible, so that content analysis would be possible and readers of this article can access a variety of instruments.

Even though no psychometric criteria were specified, we decided to include reliability estimates of the instruments for interested readers. However, we would like to mention that retest-reliability estimates should be considered with caution, as TA is proposed to be dynamic in it’s nature (e.g., due to temporary conflicts, see also Safran et al., 2011; Lilliengren et al., 2015).

##### 2.1.1.1 Drawing upon past reviews

**Figure 1:**
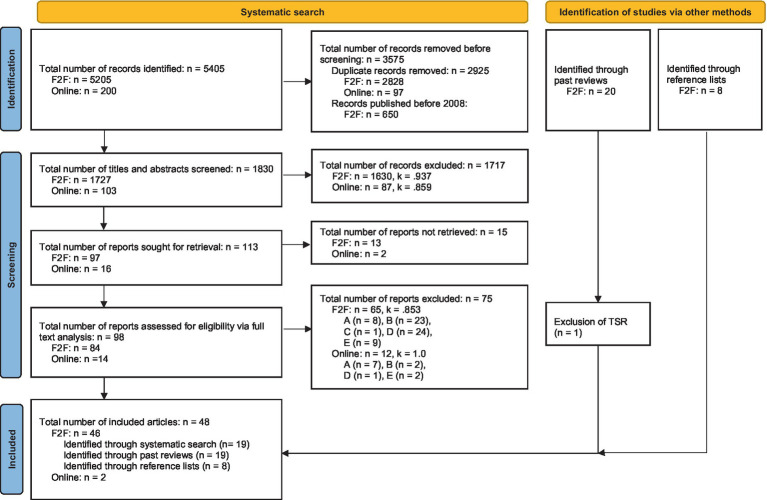
Flowchart of the search for measures of TA in F2F- and Online-PT. A, did not meet inclusion criteria for population (voluntary, individual adults) or setting (not F2F- or online-PT, respectively); B, did not meet inclusion criteria for construct (not TA, only a part of TA or more constructs than only TA are measured); C, did not meet inclusion criteria for instrument type (no self-report-measure); D, instrument already included; E, No instrument presented; F2F-PT, face-to-face-psychotherapy; PT, psychotherapy; TA, therapeutic alliance; TSR, therapy session report. Template from Page et al. (2021).

Our starting point were the reviews by Elvins and Green (2008) and by Ardito and Rabellino (2011). Here, we identified 20 instruments that met the inclusion criteria. However, the detailed research concerning the Therapy Session Report (TSR) (Orlinsky and Howard, 1967; Orlinsky et al., 1975) revealed that this instrument was originally developed for measuring different facets of the psychotherapeutic process of which only one represented the TA (Orlinsky and Howard, 1967; Orlinsky et al., 1975). For this reason, the TSR was disregarded and the remaining 19 instruments were included based on the two existing reviews (see Figure 1).

##### 2.1.1.2 Systematic literature search

Systematic literature research was performed on February 6th, 2023 and the following search term was applied: ([“therapeutic alliance” OR “helping alliance” OR “working alliance” OR “therapeutic relationship”] AND “adult psychotherapy” AND [“questionnaire” OR “assessment tool” OR “measurement” OR “inventory”]) and searched for articles in different data bases (PubMed, MEDLINE, Web of Science, Science Direct, Scopus). We identified 5,205 articles. After removing duplicates, 2,377 articles remained. These were then filtered so that only those who were published in 2008 or later were visible. The titles and abstracts of these 1,727 articles were screened in consideration of the inclusion criteria by two independent raters (E.S. and T.S.). We selected 97 articles for whole text analysis. Of these, 13 articles could not be accessed, so that 84 full texts were assessed for eligibility. Reasons for exclusion were not meeting inclusion criteria for population (voluntary, individual adults) or setting (F2F-PT; *n* = 8); not meeting inclusion criteria for construct (not TA, only a part of TA or more than only TA are measured; *n* = 23); not meeting inclusion criteria for instrument type (self-report-measure; *n* = 1); instrument had already been included (*n* = 24); no instrument being presented in the article (*n* = 9). Finally, 19 articles were retained. The inter-rater-reliability was calculated with Cohen’s Kappa (Cohen, 1960). Disagreements between raters were resolved by discussion. Figure 1 shows the selection process and the reliability indices.

The Individual Therapy Alliance Scale revised Short Form (ITASr-SF) was retrieved in the paper by Karam et al. (2015). However, there was no complete list of items in this paper. The items can be found in the article by Pinsof et al. (2008), which is listed in Supplementary Tables S1, S2. Readers should acknowledge that the Attachment Based Alliance Questionnaire (ABAQ) (Johnson et al., 2018) was not primarily developed for the measurement of TA in individual PT, but in couples PT. However, as the respective dyadic relationships between one partner and the therapist are separately measured and because the conceptual background stems from TA in individual PT, the ABAQ was kept. Additionally, the ABAQ was the only instrument to operationalize TA from an attachment theory perspective. Attachment style plays a central role in the conceptualization of TA (e.g., Diener and Monroe, 2011; Mallinckrodt and Jeong, 2015).

##### 2.1.1.3 Articles identified via reference lists

While reading the articles retrieved via existing reviews and our own systematic search, more instruments were found. The first author of this article retrieved further instruments via reference lists. Eight instruments were identified, of which 4 were not completely new instruments, but revisions, short versions or versions for different rating perspectives of instruments that were already included in our list (Gelso et al., 2005; Kim et al., 2008; Shelef and Diamond, 2008; Cahill et al., 2012; see Supplementary Table S1).

#### 2.1.2 Therapeutic alliance in online-psychotherapy

Another systematic literature search for the identification of instruments to measure TA in adult Online-PT was performed on February 7th, 2023 in the same databases (PubMed, MEDLINE, Web of Science, Science Direct, Scopus) (key words [“therapeutic alliance” OR “helping alliance” OR “working alliance” OR “therapeutic relationship”] AND “adult online psychotherapy” AND [“questionnaire” OR “assessment tool” OR “measurement” OR “inventory”]). No publication time slot was specified. The inclusion criteria were identical to the first search with one difference: The instruments should be aimed at TA in individual adult Online-PT. A total of 200 articles were found, after removing duplicates 103 remained. After screening titles and abstracts, 16 articles were selected for whole-text-analysis of which 2 could not be accessed. In the remaining 14 articles, 2 instruments for the measurement of TA in Online-PT were identified. Reasons for exclusion were not meeting inclusion criteria for population (voluntary, individual adults) or setting (Online-PT; *n* = 7); not meeting inclusion criteria for construct (not TA, only a part of TA or more than only TA are measured; *n* = 2); instrument had already been included (*n* = 1); no instrument being presented in the article (*n* = 2). The inter-rater-reliability was again calculated with Cohen’s Kappa (see Figure 1).

### 2.2 Qualitative content analysis

The analysis was conducted by two independent raters using the MAXQDA program (VERBI Software, 2021) and was based on the recommendations of Kuckartz and Rädiker (2022) for content-structuring qualitative content analysis. First, all items and descriptors of the instruments were loaded into the program. Taken together, we imported 1,014 items and descriptors measuring TA. Items from the Therapeutic Alliance Rating System (TARS) (Marziali et al., 1981) and Vanderbilt Therapeutic Alliance Scale (VTAS) (Marmar et al., 1986) were not accessible. Also, only 11 out of 30 items could be accessed from the Kim Alliance Scale (Kim et al., 2001); likewise 12 out of 30 possible descriptors could be accessed of the Psychotherapy Status Report (PSR) (Frank and Gunderson, 1990). These instruments are thus not or not fully included in the qualitative content analysis. Both raters first read the items, highlighted important paragraphs and took notes to familiarize themselves with the material. Then, three main categories were developed together deductively: The category “neutral terms” should include all items that referred to interactional phenomena or general characterizations of TA (e.g., aspects of unity, cooperation, reciprocity, atmosphere, …). All items that only addressed patient characteristics, skills, behaviors or feelings (e.g., active cooperation, trust toward therapist, …) should be sorted into the category “patient-related terms” (hereafter “patient terms”). Similarly, all items that addressed therapists’ characteristics, skills, behaviors, or feelings (e.g., competence, authenticity, …) should fall into the category “therapist-related terms” (hereafter “therapist terms”).

The first rater sorted all items into the three categories while inductively creating subcategories that each represented a different content aspect of the items. One item could contain one or more content aspects and therefore be coded repeatedly. To ensure objectivity, there was no repolarization of negative aspects. Good and bad cooperation, for example, were coded as two separate subcategories and not as two manifestations of one dimension. The final product was a category system containing all items. Finally, the finished category system was revised by the first rater. Now the second rater joined in and both raters independently re-sorted all items into the revised system.

After coding the first 10 instruments, the raters performed a manual comparison (“consensual coding”) of their coding of the items; in addition, possible ambiguities in the coding process were clarified. Each item was checked individually to what extent it was sorted into the same subcategory(ies) by both raters. After this, all other instruments were analyzed. Finally, percentage of agreement was calculated as a measure of inter-coder-reliability across all instruments. This indices gives information about the agreement on the presence of a subcategory in an instrument which the MAXQDA manual recommends when dealing with relatively short texts and working with many codes (VERBI Software, 2020).

Short versions of some instruments were available in addition to the original versions. In this case, we examined whether the short versions contained only an excerpt of the original items and, consequently, whether an analysis of the original version would be exhaustive in terms of content. This was the case for the ARM-5 and ARM-12, as well as for the various short and online versions of the WAI. Thus, these questionnaires were not processed within the framework of the qualitative content analysis. However, if different perspectives (patients, therapists, observers) were available for an instrument, all versions were analyzed because their items did not always correspond exactly in terms of content (e.g., RRI for therapists or clients: RRI-T and RRI-C, respectively, Gelso et al., 2005; Kelley et al., 2010).

## 3 Results

### 3.1 Overview of instruments

All three searches together provided 46 instruments for TA in F2F-PT (see Figure 1). Which instrument was identified by which search can be retraced in Supplementary Table S2. For TA in online-PT, 2 articles could be found (Figure 1). All instruments including their conceptual backgrounds, characteristics and reliability estimates are summarized in Supplementary Table S1. A detailed and narrative presentation of the conceptual backgrounds from different author groups can be found in section 3.3.

Concerning the instruments for TA in F2F-PT, 24 instruments operationalize one or more theoretical conceptualizations of TA (AROS; ANS; ABAQ; BQTA; CRF; HAQ-1; HAQ-2; HAcs; HAr; Menninger Alliance Scales; RRI-C; RRI-T; RRQ; RDFS; RI; 3RS; SRS; WAI; WAI-T; WAI-S; WAI-S-R; S-WAI-O; BAI; SAI, please consider Supplementary Table S1 for full names and references). Of these, Bordin (1979) conceptualization is operationalized most frequently, followed by the conceptualization of Safran and colleagues (Safran and Muran, 2000; Safran et al., 2002). Furthermore, 14 instruments are a synthesis or an adaptation of existing measures (ARM; ARM-5; ARM-12; Brief CALPAS; CALPAS; CEI; ITASr-SF; KAS-R; STA-R; TARS; TBS; VPPS; VTAS; VTAS-R). Note that the items of the Brief California Psychotherapy Alliance Scales (Brief CALPAS) were assembled based on personal recommendation and not on psychometric criteria. There are three instruments based on literature searches alone or combined with the operationalization of theoretical conceptualizations (4PAS, AiA, PRQ). Three instruments are based on or at least incorporated an empirical survey of staff and patients (IRS; KAS; STAR). The HAS and PSR provide no information on their conceptual background. Concerning the instruments for TA in Online-PT, both identified measures (WAI-I; WAI-TECH-SF) were an adaptation of the Working Alliance Inventory (WAI) (Horvath and Greenberg, 1989) that could be applied in therapist-guided Online-PT.

### 3.2 Qualitative content analysis

The revised category system consisted of 17 subcategories within the neutral terms, 29 subcategories within the patient terms, and 32 subcategories within the therapist terms, leading to a total of 78 subcategories reflecting different content aspects of TA. The average percentage of agreement across all instruments was 78.69%. This means that on average 78.69% of the subcategories used in one instrument were identical between the two raters. No cut-off values are provided as a guide for interpreting agreement coefficients, as the level of agreement also depends on the number of subcategories and categories in a system (Rädiker and Kuckartz, 2019). Supplementary Table S3 gives the percentage of agreement per instrument as well as overall. The five most frequently used subcategories were: 1. “self-disclosure, authenticity” (patient-terms) with 55 total codings, e.g., the item “I was open and honest with my therapist” (RRI-C) (Kelley et al., 2010); 2. “Agreement on problem-causes, goals and methods” (neutral category) with 53 codings, e.g., “My patient and I are working on common goals” (BQTA) (Widschwendter et al., 2016); 3. “Active participation, motivation, compliance” (patient-terms) with 52 codings, e.g., “Did you take initiative in bringing up the subjects that were talked about?” (TBS) (Saunders et al., 1989); 4. “Trust, secure attachment” (patient-terms) with 52 codings, e.g., “I feel I can depend upon my therapist” (HAQ-1) (Eich et al., 2018) and 5. “Not taking needs/abilities/wishes of patient into account” (therapist-terms) with 49 codings, e.g., “My therapist is inflexible and does not take my wants or needs into consideration” (ANS) (Doran et al., 2012). The last subcategory seems to reflect an aspect of harmful TA, but the sixth most frequently used subcategory was “Taking needs/abilities/wishes of patient into account” (therapist-terms) with 48 codings, e.g., “My therapist checked with me to see if there were other concerns that we needed to address” (AiA) (Owen et al., 2013). All categories and subcategories with their frequency of usage and exemplary items is provided in Supplementary Table S4.

### 3.3 Overview of conceptualizations of therapeutic alliance

#### 3.3.1 Psychodynamic roots

The roots of the TA-concept are found in psychoanalysis, where Freud (1982a) described the role of transference. He further describes how analysts can establish a strong, positive transference through sympathetic understanding, serious interest, and a supportive attitude (Freud, 1982b; Horvath and Luborsky, 1993). Later, Freud postulates that establishing a relationship grounded in reality is necessary for healing (Horvath and Luborsky, 1993). This means that positive transference or even idealization of the therapist are not sufficient conditions for healing (Horvath and Luborsky, 1993). Previously, Sterba (1934) had also spoken of an alliance between the therapist and the patient’s rational parts, which should enable the latter to benefit from the therapist’s interpretations in a self-reflective way. Therapists can promote the alliance, for example, by using the word “we” (Sterba, 1934; Hougaard, 1994).

Zetzel (1956) picks up on the last point and specifically labels alliance, as opposed to transference, as the non-neurotic component of TA which allows patients to follow the therapist’s interpretations and reflect on the present relationship as well as past relationships.

Greenson (1965) builds on the distinction between neurotic transference and the therapeutic working relationship, coining the term “working alliance” (Horvath and Luborsky, 1993). At the same time, he emphasizes that the rational and reflective aspects are not to be seen separately from the transference aspects, since the latter are to be analyzed with the help of the former (Greenson, 1965). According to Greenson (1967), TA consists of three components: the transference, the working alliance (which is comparable to Zetzel’s alliance) and the real relationship, which is later taken up by Gelso and Carter (1985, 1994) and Gelso et al. (2005). The real relationship means the personal relationship aside from the professional roles (Hougaard, 1994; Gelso et al., 2005). Accordingly, the real relationship exists independently of the work aspect, although there is a mutual influence. In contrast to transference, the real relationship is grounded in reality and contains two parts: the authenticity of patient and therapist (“genuiness”) and the respective realistic, undistorted perception of the other person (“realism”; Gelso and Carter, 1994; Gelso et al., 2005).

Frieswyck et al. (1984) define the therapeutic alliance exclusively through the patient’s active collaboration. The authors thus strive to distinguish both the patient’s relational experiences (the transference) and the therapeutic techniques (therapist techniques) from the therapeutic alliance. This distinction is intended to avoid confusing TA with the therapist’s personality or competence, or with specific interventions, so that one can empirically investigate these very relationships (Allen et al., 1984; Frieswyck et al., 1984).

Sexton and Whiston (1994) draw on the work of Gelso and colleagues and consider the three components of TA from a social constructivist perspective. That is, they are co-constructed in a specific context through an interactional process by patient and therapist, and are not determined by patient or therapist alone. For example, the therapist’s empathy must be perceived by the patient, and the patient’s transference must be interpreted accordingly by the therapist (see also Rogers, 1957, section 3.3.3).

As can be retraced from Supplementary Table S1, instruments that incorporate one or more of these approaches include the ARM, CALPAS, Menninger Alliance Scales, PRQ, RRI, STA-R, TARS, and VTAS. However, most of these instruments reassemble existing items from various scales, so that they do not purely reflect the sources mentioned above.

#### 3.3.2 Attachment-based conceptualizations

Bowlby (2012) applies attachment theory to psychotherapy and presents corresponding clinical implications. In line with psychoanalytic transference, he assumes that past relationship experiences as well as attachment style can be transferred to TA and that mistrust, anger, fear of loss, idealization, and other phenomena can become manifest (Diener and Monroe, 2011; Bowlby, 2012). Bowlby (2012) narrows the essential task of psychotherapy down to exploring and restructuring the patient‘s attachment style, which is to be done through both cognitive understanding and corrective emotional attachment experiences. The patient‘s sense of security in TA, as well as their increasing ability to establish a need-satisfying relationship with the therapist, receive particular emphasis in attachment-oriented conceptualizations of TA (Horvath and Luborsky, 1993; Diener and Monroe, 2011; Johnson et al., 2018). The ABAQ (see Supplementary Table S1) operationalizes this approach.

#### 3.3.3 Theories of therapist variables

In his client-centered concept of TA, Rogers (1957) establishes the necessary and sufficient conditions for therapeutic personality change, including the three Therapist-Offered Conditions (TOC) empathetic understanding, congruence, and unconditional acceptance of the patient. Another condition is the patient’s perception of these offerings—Horvath and Luborsky (1993) even conclude that the perceived empathy is more important than the actual therapist behavior.

Strong (1968) presented a conceptualization of TA as a social influence process (Horvath and Luborsky, 1993). He suggested that the expertness, attractiveness and trustworthiness of the therapist as perceived by the patient would increase the therapist‘s interpersonal influence and thus therapeutic success (Strong, 1968; Barak and LaCrosse, 1975).

The RI and CRF are based on these considerations, respectively (see Supplementary Table S1).

#### 3.3.4 Systemic view

Pinsof and Catherall (1986) and Pinsof et al. (2008) view TA from a systemic perspective, referring to it as the collaborative aspect of the relationship (Pinsof and Catherall, 1986). In their “Integrative Psychotherapy Alliance” model (IPA) (Pinsof and Catherall, 1986), there are two domains of the therapeutic alliance: The content domain means the agreement on goals and tasks of therapy as well as the bond between patient and therapist (see section 2.5 for Bordin, 1979 conceptualization). The Interpersonal System domain specifies the people involved in the respective TA (e.g., dyadic relationship between the therapist and the patient; dyadic relationship between the therapist and the patient’s partner; relationship between the therapist and the patient’s friend group including the patient herself). An instrument that is built upon the systemic view is the ITASr-SF (see Supplementary Table S1).

#### 3.3.5 Pantheoretical conceptualizations

Variables common to all psychotherapy methods could explain the so-called “Dodo-bird verdict” (Tschacher et al., 2014; Pfammatter and Tschacher, 2015; Horvath, 2018); that is, they could explain why different therapy methods are similarly effective (Horvath and Luborsky, 1993; Ardito and Rabellino, 2011). TA is conceptualized as such a pantheoretical factor (Horvath and Luborsky, 1993; Ardito and Rabellino, 2011).

Luborsky (1976) distinguishes between two “helping alliances”: Type 1 is evident especially at the beginning of therapy and is based on the experience of a supportive and helpful therapist. Type 2 describes the collaborative and productive aspect of a working relationship in which both parties share responsibility for the achievement of goals (Luborsky, 1976; Horvath and Luborsky, 1993). According to Luborsky (1976), the Type 2 alliance is associated with long-term success, as it reflects the patient’s ability to apply the skills acquired in therapy autonomously. The Penn Scales (HAcs, HAr, HAQ-1, HAQ-2) operationalize the Type-1 and -2 alliances and the VTAS also includes Luborsky (1976) considerations.

Bordin (1979, 1983) identifies three factors of his “working alliance”: 1. The agreement on goals (What should be achieved?), 2. The agreement on tasks (How should these goals be achieved?) and 3. The development of a personal bond between patient and therapist (mutual positive feelings toward each other, that are required to sustain a particular collaboration). This conceptualization is the most commonly used (Doran, 2014). The WAI and it’s revised and short forms are based on Bordin (1979), but many more instruments at least incorporate this approach.

Safran’s research group (Safran and Muran, 2000; Safran et al., 2002) emphasizes the role of negotiation of the patient’s and therapist’s respective needs, views, and goals. Both have to find out to which extent they can compromise. Safran and colleagues build on later work by Bordin (e.g., Bordin, 1983), in which the resolution of conflicts has been proposed as a central aspect of TA (Safran et al., 1990; Doran et al., 2012). The authors present three types of conflicts (“ruptures”): Disagreement regarding the goals of therapy, disagreement regarding the tasks in therapy, and ruptures in the bond between patient and therapist. These conflicts can arise from patients’ past relational experiences, which in turn provoke counter-reactions in therapists (Safran and Muran, 2000). Corresponding instruments are the RRQ, 3RS, ANS, and AROS (see Supplementary Table S1).

Mearns and Cooper (2005, 2018) add relational depth as an important aspect to describe TA. “Relational depth” is defined as a state of profound contact and engagement between people that can occur in individual moments as well as characterize the entire relationship (Mearns and Cooper, 2018, Preface p. xvii). The RDFS (see Supplementary Table S1) is based on this approach.

#### 3.3.6 Empirical approaches and syntheses

Fiedler (1950) presented 119 sentences describing TA to therapists from different schools, which they were asked to evaluate. He concluded that there was a similar conception of the ideal TA across different schools. Anderson and Anderson (1962) continued the empirical testing of the ideal TA and developed the Interview Rating Scale (IRS), which operationalized the definition of the ideal relationship as well as effective communication and was later used for developing the Counseling Evaluation Inventory (CEI) (Linden et al., 1965) again. This strand was further pursued by Orlinsky and Howard (1978, 1986): the authors synthesized results of empirical research and postulated the following three dimensions of TA: 1. role-investment, 2. empathetic resonance and 3. mutual affirmation. Role-investment is relabeled “working alliance” in later work (Saunders et al., 1989; Elvins and Green, 2008) and refers to the investment of patient and therapist in the therapy process as well as their motivation. Empathic resonance describes the feeling of being “on the same wavelength” as well as mutual trust. Mutual affirmation represents concern for each other’s well-being and is related to Rogers (1957) unconditional positive regard (Orlinsky and Howard, 1986; Saunders et al., 1989; Elvins and Green, 2008). Orlinsky et al. (1975) had previously identified seven bipolar factors of the shared therapy experience using their Therapy Session Report (TSR) (Orlinsky and Howard, 1967; Orlinsky et al., 1975), of which the seventh factor was TA. The TSR represents an important starting point for the development of other instruments [e.g., Therapeutic Bond Scales (TBS); Saunders et al., 1989]; Vanderbilt Psychotherapy Process Scale (VPPS) (Strauß, 1991).

An early overview of various conceptualizations is provided by Hougaard (1994). Consequently, an outline of a generic model of TA is presented that includes two components: The personal relationship, or socio-emotional aspects (“personal relationship area consisting of the socio-emotional aspects”) and the collaborative relationship, or task-related aspects (“collaborative relationship area consisting of the task-related aspects,” Hougaard, 1994, p. 70).

## 4 Discussion

After conducting a systematic literature search for measures of TA in individual Online- and F2F-psychotherapy with adults, we analyzed the conceptual backgrounds of the final 48 instruments and performed a qualitative content analysis. This allowed us to extract the main aspects of TA reflected by current measures: Instead of over 1,000 items and descriptors, we can provide an overview of 78 categories that represent the content of the measures.

Most instruments operationalize one or more theoretical conceptualizations of TA or recombine items from existing measures. Of the theoretical conceptualizations, Bordin (1979) and the Safran group (Safran and Muran, 2000; Safran et al., 2002) are operationalized most frequently in measures of TA in F2F-PT. Both measures of TA in Online-PT relied on Bordin (1979) conceptualization. Hence, the aspects of TA that should be most frequently emphasized in the current literature are the formation of a bond, the agreement on goals and tasks in therapy and the occurrence and resolution of conflicts between patients and therapists. This generally matches with our qualitative content analysis of the items, which revealed that self-disclosure and authenticity of the patient, agreement between patient and therapist, the patient’s active participation, motivation and compliance as well as his*her trust and secure attachment to the therapist and the therapist’s consideration of the patient’s needs, abilities and wishes seem to be the five most widely used aspects of TA in current measures. Self-disclosure and trust of the patient as well as the therapist’s consideration of the patient’s needs, for example, seem to be of fundamental importance for the formation of an emotional bond and for openly addressing and resolving conflicts. Also, Bordin’s agreement-component is reflected directly in our subcategories. The active participation and motivation of the patient, however, is not directly addressed in Bordin’s or Safran et al.’s conceptualization, but can be found in other theories, e.g., in Luborsky (1976) helping alliance.

While theoretical approaches on TA are considered regularly, the experience of psychotherapists and the opinions of patients are often neglected when defining TA. This could become a problem, if psychotherapists and their patients regard other aspects as central for the formation of TA, which would lead to a divergence between the current, mostly theoretical, conceptualizations and the empirical conceptualization of TA. Only three measurements are based on an empirical survey: The Interview Rating Scale (IRS) (Anderson and Anderson, 1962) is based on the empirical data from Fiedler (1950), however this data is now almost 75 years old. The Kim Alliance Scale (KAS) (Kim et al., 2001) and the Scale to Assess the Therapeutic Relationship (STAR) (McGuire-Snieckus et al., 2007) are the only identified measures that used a bottom-up-approach for developing items and implemented an empirical survey of practicing clinicians in this millennium. Still, only medical staff participated in the item development for the KAS and the STAR is based on the survey of social workers, nurses, psychologists, one occupational therapist and patients. An empirical conceptualization and operationalization of TA in individual PT with adults remains yet to be undertaken. Additionally, those instruments which were developed with a bottom-up-approach are rarely used: According to recent meta-analyses, the most widely used instruments are the WAI with a percentage of 69%, followed by the California Psychotherapy Alliance Scales (CALPAS) (Gaston and Marmar, 1993) HAQ and Vanderbilt Psychotherapy Process Scale (VPPS) (Strauß, 1991; Flückiger et al., 2018; Del Re et al., 2021). A possible explanation could be that the empirically developed instruments are not explicitly constructed with the help of a psychotherapeutic sample, which emphasizes the need for this approach even more.

Even though no instrument or model of TA was developed following these studies, there have been attempts to empirically conceptualize TA: Bachelor (2013) surveyed psychotherapy patients and therapists about their personal definition of TA by letting them complete items from three different questionnaires (WAI, HAQ, CALPAS). She then conducted two separate factor analyses for patients and therapists each. The author concluded that the empirical perceptions differed significantly from the theoretical conceptualizations of TA and that therapists and patients also identified partially different components. A criticism of this study is that items from existing operationalizations of TA were used to empirically define them. While this approach allows existing conceptualizations to be tested, the additional identification of novel components is not possible. Furthermore, the instructions for the participants are not clearly described: Were they assessing the quality of their relationship with their therapist, or were they assessing the importance of this item for characterizing general TA?

To our knowledge, only Gelo et al. (2016) have conducted a qualitative survey on TA so far. The authors asked an Italian sample of 63 psychotherapists of cognitive behavioral therapy 1. what TA is for them, 2. To name five nouns and five adjectives regarding “therapeutic relationship” and 3. To sort the latter by personal relevance. Aspects such as empathy, acceptance, or trust were most important to the psychotherapists. While the authors interpret their findings as generally consistent with theory, they do not cite any specific theory that fits their empirical findings. On closer examination, it is noticeable that especially Bordin’s components of TA (Goals, Tasks, Bond) were not mentioned, so that a discrepancy between the most frequent operationalization and the empirical understanding of TA can be noted. Like Bachelor (2013) and Gelo et al. (2016) call for the development of questionnaires that incorporate these empirically-derived aspects of TA.

The integration of personal, practical experience and expertise concerning the important aspects of TA from psychotherapists and patients seems to be overlooked frequently when conceptualizing and operationalizing TA. The development of an empirically-founded and metatheoretical conceptualization of TA and an according measure should be the target of future research.

Despite the growing interest in Online-PT, our search only yielded two instruments for TA in this context. Both build upon Bordin (1979) conceptualization. The lack of explicit conceptualizations of TA in Online-PT could be explained because the same measures are often used for measuring TA in Online- and F2F-PT (e.g., Reynolds et al., 2007; Bisseling et al., 2019; Vernmark et al., 2019; Duffy et al., 2020; see also reviews by Wehmann et al., 2020; Kaiser et al., 2021). However, it seems troublesome to simply apply the same conceptualizations of TA that are used in F2F-PT (e.g., Bordin) to Online-PT. First, the exact format of Online-PT should be defined: Maybe large portions of traditional conceptualizations can be transferred to psychotherapy with a human therapist over phone and video calls. However, we would expect TA with a chatbot or an app to differ fundamentally. For example, the conflicts and their resolution emphasized by the Safran group could serve as a corrective experience in person-to-person-relationships and strengthen TA in F2F-PT, whereas a conflict with an app or a program could be purely frustrating or even lead to discontinuation of PT with no chance of resolution. Also, we would anticipate the emotional experience behind Bordin (1979) bond-aspect of TA to differ qualitatively between human relationships and human-computer-interaction. This gives rise to the question whether Bordin’s conceptualization can be applied to the TA between a patient and an online-program, as the WAI-TECH-SF and WAI-I currently do (see Supplementary Table S1).

Regarding the broad variety of conceptualizations (see section 3.3), the amount of according operationalizations (see Supplementary Table S1) and our extensive list of 78 content aspects in the items of the instruments, it becomes evident that conducting coherent research is difficult. Flückiger et al. (2018) already addressed this lack of a “precise consensual definition” (p. 318). The authors conclude that the 39 different measures of TA used in the studies of their meta-analysis “overlap to some extent, but do not share a common point of reference” (p. 318). This conceptual diversity also manifests in inconsistencies concerning the convergent validities of the instruments: Some authors report a high correlation between measures (e.g., Martin et al., 2000; Di Malta et al., 2019), while others report or criticize a low correlation (e.g., Fenton et al., 2001; Eubanks et al., 2018; Horvath, 2018) and a third group expresses inconsistent findings (e.g., Tichenor and Hill, 1989; Gelso et al., 2005). Several conceptual challenges have been discussed elsewhere (Flückiger et al., 2018; Horvath, 2018).

One conceptual imprecision that seems particularly important and has not been addressed sufficiently in previous works concerns the distinction of TA from attachment style. The general attachment style of patients, which already exists before starting psychotherapy, seems to have an impact on TA (e.g., Strauß and Schwark, 2007; Mallinckrodt and Jeong, 2015; Mallinckrodt et al., 2017). A conceptual imprecision however is the distinction between TA and the patient’s particular attachment style to the therapist, which has been much discussed (e.g., Strauß and Schwark, 2007; Obegi, 2008; Lilliengren et al., 2015; Mallinckrodt and Jeong, 2015; Mallinckrodt et al., 2017; Kim, 2018; Petrowski et al., 2019). Attachment style to the therapist can be assessed separately, e.g., using the Client Attachment to Therapist Scale (CATS) (Mallinckrodt et al., 1995) or the Personal Attachment to Therapist Questionnaire (PATS) (Bar-El and Gil, 2022). However, as described in section 3.2.2, there are also conceptualizations of TA from an attachment perspective, with secure attachment to the therapist operationalized as a central component of TA (Bowlby, 2012; Johnson et al., 2018). There are findings that suggest the two concepts to be nearly interchangeable: Mallinckrodt and Jeong (2015) find strong correlations of *r* = 0.76 between secure attachment (as measured by the CATS) and the WAI score in their meta-analysis, Yotsidi et al. (2018) analogously report a correlation of *r* = 0.82, and Bachelor et al. (2010) also report a correlation coefficient of *r* = 0.77 between a secure attachment style to the therapist and TA. These correlations are higher than many correlations between two measures of TA (i.e., higher than convergent validity; e.g., *r* = 0.75 in Doran et al., 2012; *r* = 0.64 in Munder et al., 2010; *r* = 0.47 in Gelso et al., 2005; no significant correlation in Eubanks et al., 2018). Moreover, Mallinckrodt et al. (2017) find that the correlation between secure attachment to therapist and therapy outcome is almost exactly the same as the correlation between WAI score and therapy outcome (respectively, *r* = 0.274; *r* = 0.275). The additional value of TA for predicting therapy success could thus be questioned. On the other hand, the correlation between attachment style to therapist and TA depends on the exact attachment style: for example, in Bachelor et al. (2010) and Yotsidi et al. (2018), secure attachment had a positive effect and avoidant-fearful attachment style had a negative effect on TA; however, there was no correlation between the preoccupied-merger attachment style and TA. Obegi (2008) suggests that attachment to the therapist is mainly important for Bordin’s “bond” component of TA, but adds that it is too a prerequisite for patient self-opening and collaboration. Lilliengren et al. (2015) expect attachment style to remain stable while TA is dynamic. The authors argue that good TA can exist without a secure attachment to the therapist (e.g., in conscious collaboration) and temporary negative TA can be reported even with a secure attachment to the therapist (e.g., in conflict). Allison and Rossouw (2013) argued from a neurobiological perspective that security in TA is created by satisfying the needs for attachment and control in patients and that this is needed for behavioral and emotional interventions. Kim (2018) discusses the mutual influence of attachment, TA and the therapist’s empathy and furthermore presents distinct and common brain areas for each of these three constructs. Kim (2018) also concludes that therapist empathy as well as secure attachment may be important prerequisites for TA. In conclusion, patient attachment to therapist could play an important role or even base for TA, however additional distinct aspects of TA remain. Future studies should address this conceptual differentiation and quantify to which degree TA and attachment to the therapist are interchangeable with regard to treatment outcome. Possibly there are certain patients that require TA to have an attachment focus, whereas other patients profit from a more psychoeducative, task- and goal-oriented TA.

An empirical survey of patients and psychotherapists concerning their definition of TA, as mentioned above, could also provide valuable information and help to clarify these conceptual imprecisions. Moderators such as attachment style should be included.

The various attempts to conceptualize and measure TA show the immense complexity and the many aspects of this construct. Future studies should consider to which extent language can provide exhaustive information when defining a human relationship. Phrases such as “being on the same wavelength” (Saunders et al., 1989) show that not every feeling concerning TA can be adequately put in words. In line with this, nonverbal indicators of TA such as interpersonal synchrony could provide valuable additional information in characterizing a relationship that is beneficial to therapy success (see Ramseyer and Tschacher, 2011; Ramseyer and Tschacher, 2014; Wiltshire et al., 2020; Zilcha-Mano et al., 2020; Mende and Schmidt, 2021; Meier and Tschacher, 2023). It remains to be investigated, if nonverbal indicators of TA demonstrate higher convergent validities among each other as well as with traditional linguistic measures of TA.

## 5 Limitations

It remains to be noted that only articles published in 2008 or later were included in the systematic literature search for measures of TA in F2F-PT. Because of this, 650 articles were discarded. For articles that were published before 2008, we relied on the work of the existing reviews (Elvins and Green, 2008; Ardito and Rabellino, 2011) and integrated the instruments that were given there. We chose to include the years 2008, 2009, and 2010 in our analysis, although the review from Ardito and Rabellino was published 2011 and already covered these years. We found two further instruments in this time frame (Misdrahi et al., 2009; Kelley et al., 2010).

The search for measures of TA in F2F-PT was more extensive than the corresponding search for Online-PT. The former consisted of drawing upon past reviews, performing a new systematic literature search as well as a search of reference lists while the latter was limited to a systematic literature search. Since no reviews for the measurement of TA in Online-PT were found, the first part could not be equally implemented. This could be due to the fact that Online-PT is relatively new and no reviews have yet been conducted, or because the same measures are often used for measuring TA in Online- and F2F-PT. While looking for reviews, no other instruments to measure TA in Online-PT were found, hence a further search was dropped.

## 6 Conclusion and further research

There is a broad variety of instruments for measuring TA in adult PT. These instruments oftentimes operationalize theoretical conceptualizations of TA from different author groups, of which there is a wide spectrum as well. Practicing psychotherapists and patients are not included in the conceptualization or operationalization sufficiently so that their valuable expertness is not represented in models or measures. The amount of theories and measures leads to conceptual imprecisions regarding TA. A pantheoretical and empirical conceptualization of TA and the development of a corresponding valid measure that encompasses the experience of therapists and patients could provide a possible solution for these problems. When surveying patients and therapists, qualitative methods and moderators such as attachment style should be included. Furthermore, nonverbal indicators of TA should complement the conceptualization.

## Data availability statement

The raw data supporting the conclusions of this article will be made available by the authors, without undue reservation.

## Author contributions

ES: Conceptualization, Data curation, Formal analysis, Methodology, Writing – original draft, Writing – review & editing. TS: Data curation, Formal analysis, Methodology, Writing – review & editing. AP: Project administration, Writing – review & editing. MS: Conceptualization, Methodology, Writing – review & editing. SL: Conceptualization, Methodology, Project administration, Supervision, Writing – review & editing.
