# Supplementary material for: Therapeutic alliance in individual adult psychotherapy: a systematic review of conceptualizations and measures for face-to-face- and online-psychotherapy
Source: Front Psychol. 2024 Jun 27;15:1293851. doi: 10.3389/fpsyg.2024.1293851 (PMC11238262; doi:10.3389/fpsyg.2024.1293851)
Supplement: Supplementary file 1 [file Data_Sheet_1.docx]

Supplementary Material

Therapeutic Alliance in Individual Adult Psychotherapy: A Systematic Review of Conceptualizations and Measures for Face-to-Face- and Online-Psychotherapy

Saxler, Eva*; Schindler, Theresa; Philipsen, Alexandra; Schulze, Marcel; Lux, Silke

*** Correspondence:** Eva Saxler: [evasaxler@web.de](mailto:evasaxler@web.de); Silke Lux: [silke.lux@ukbonn.de](mailto:silke.lux@ukbonn.de)

# Figures and Tables

# Table S1: Measures of Therapeutic Alliance with According Backgrounds and Characteristics

| Instrument | Source with access to items | Conceptual backgound | Description and scales | Number of items and response format | Pers-pective | Reliability estimates |
| --- | --- | --- | --- | --- | --- | --- |
| 4-Point ordinal Alliance Scale (4PAS) | Misdrahi et al., 2009 | Literature search for measurements of TA, e.g. Luborsky (1983). | 2 factors: „empathy experienced“ and „psychoeducation“. | 11 items;  4-point-likert-scale;  Global rating of TA on a 8cm long visual analog scale | P | Internal consistency: Cronbach’s α = .91 |
| Agnew-Davies Relation-ship Measure (ARM); | Agnew-Davies et al., 1998; | Pantheoretical conceptualization of TA based on several reviews (Gaston, 1990; Gelso & Carter, 1994; Horvath & Luborsky, 1993; Sexton & Whiston, 1994). Item development via integration and factor analysis of existing scales (not further specified). | 5 scales: „Bond“, „Partnership“, „Confidence“, „Openness“ and „Client Initiative“. | 28 items;  7-point-likert-scale | P, T | α = .55-.87 (dependent on subscale and perspective) |
| ARM-5 und ARM-12 | Cahill et al., 2012 | Short versions of the ARM | ARM-12: 2 scales: 1.„Core-Alliance-Scale“consisting of items from the Bond-, Partnership- and Confidence-scale; and 2. „Openness-Scale“;  ARM-5: Items from Bond-, Partnership- and Confidence-Scale. | 12 or 5 items, respectively, from the long version. | P, T | ARM-12: α = .73-.89 (dependent on subscale and sample)  ARM-5: α = .64-.82 (dependent on sample) |
| Alliance and Rupture Observa-tion Scale (AROS) | Holmqvist Larsson et al., 2019; items can be inquired from the first author. | Based on Bordin (1979) and Safran et al. (2011). Bordin (1979) identifies 3 factors of TA: agreement on goals, agreement on tasks and development of a personal bond. The occurence and resolution of conflicts is regarded as a central aspect of TA by Safran and collegues (Safran et al., 1990; Safran & Muran, 2000; Safran et al., 2002; Safran et al., 2011). | 4 aspects: „Collaboration“, „Agreement“, „Mutuality“ „Genuine Concern“. | One therapy session is divided into short segments (2 min.).  The 4 aspects are rated on a 7-point-likert-scale each. 1 and 2 points are coded as ruptures and further divided into withdrawal, confrontation, misunderstanding or no signs of this aspects at all. | O | Interrater-relaibility: ICC = .81 (.77-.85)***  α = .95 |
| Alliance in Action (AiA) | Owen et al., 2013 | Bordin‘s (1979) conceptualization; additional literature search for therapist behaviors that foster a good TA. | 4 scales: „Relationship Monitoring“, „Negative Collaboration“, „Goal Checking“ and „Process Monitoring“. | 15 items;  7-point-likert-scale | P | α = .72-.76 (dependent on subscale) |
| Alliance Negotia-tion Scale (ANS) | Doran et al., 2012 | Based on Safran & Muran (2000). | 2 factors: 1. „comfort with negative feelings“; 2. „flexible and negotiable stance“. | 12 items;  7-point-likert-scale | P | α = 84 (.81-.86)*** |
| Attach-ment Based Alliance Question-naire (ABAQ) | Johnson et al., 2018;  Originally developed for couples-PT | Conceptualization of TA from an attachment perspective: Based on Bordin (1979), Bowlby (1988/2012), Bartholomew & Horowitz (1991);  Modification of items of the „Experiences in Close Relationships Scale“ (Brennan et al., 1998). Bowlby (1988/2012) assumes that past relationship experiences and attachment style influence TA (Bowlby, 1988/2012; Diener & Monroe, 2011). A secure TA that fulfills the patient’s needs is emphasized (Diener & Monroe, 2011; Horvath & Luborsky, 1993; Johnson et al., 2018). | Unifactorial structure of the „attachment-based alliance“ that differentiates between a secure and insecure attachment to the therapist. | 12 items;  7-point-likert-scale | P | α = .88 |
| Brief Question-naire on Thera-peutic Alliance (BQTA) | Widschwendter et al., 2016 | Bordin‘s (1979) conceptualization, focus on TA with chronically ill and medicated patients (e.g. schizophrenic patients). | Items represent Bordin‘s (1979) 3 factors. Patients estimate the answer of their therapists and vice versa. | 5 items;  4-point-likert-scale | P, T | α = .68-.77 (dependent on perspective) |
| California Psycho-therapy Alliance Scales (CALPAS) | Gaston & Marmar, 1993  Versions for group- and child-PT | CALPAS are the revised CALTARS (Ardito & Rabellino, 2011; Gaston, 1990). The latter are derived from the TARS (Marziali et al., 1981; Marziali, 1984) (Gaston, 1990; Ardito & Rabellino, 2011). Gaston & Marmar (1993) list different theroretical foundations of the CALPAS: several works of Freud, Sterba (1934), Greenson (1965), Bordin (1979) and Rogers (1957). | 4 scales: „Patient Working Capacity“, „Patient Commitment“, „Working Strategy Consensus“ and „Therapist Understanding and Involvement“. | 24 items;  7-point-likert-scale  Additionally, there are short versions with 12 items. | P, T, O | α = .84 (.43-.73; Gaston, 1991)*** |
| Brief CALPAS | Daniels & Wearden, 2011 | Item-assembly based on the recommendation of C. Marmar. |  | 5 items  11-point-likert-scale | P | NA |
| Counseling Evaluation Inventory (CEI) | Linden et al., 1965 | Based on the IRS by Anderson & Anderson (1962). | 3 scales: „Counseling Climate“, „Counselor’s Comfort“ and „Client’s Satisfaction“. | 21 items;  5-point-likert-scale | P | Retest reliability (14-day-interval): r = .83 |
| Counselor Rating Form (CRF) | Barak & LaCrosse, 1975 | Strong‘s (1968) conceptualization of TA as an interpersonal influence process: The client’s perception of the counselor’s expertness, trustwothiness and attractiveness increases the latter’s influence and therapy success (Barak & LaCrosse, 1975; Strong, 1968). | 3 dimensions: „Expertness“, „Trustworthiness“ and „Attractiveness“. | 36 pairs of opposite words to describe the therapist;  Bipolar 7-point-likert-scale | P, O | NA |
| Penn Scales | Helping Alliance Counting Signs (HAcs; Luborsky et al., 1983) | Luborsky‘s (1976) type-1- and type-2-alliance: Type 1 mainly occurs at the beginning of therapy and describes the patient’s experience of a supportive therapist. Type 2 describes the collaborative and productive aspect of the working alliance, in which both parties share responsibility for reaching goals (Horvath & Luborsky, 1993; Luborsky, 1976). | HAcs: Patient-related indicators of 7 TA-subtypes are described in positive and negative expression (4 for type-1-, 3 for type-2-alliances): Positive type 1: feeling helped by therapy or therapist, feeling of change, feeling understood, feeling optimistic; positive type 2: feeling of collaboration, similar conception about source of the problem, ability to work on problems self-reliantly.  Therapist Facilitating Behaviors Counting Signs (TFBcs): 9 therapist-related subtypes of TA are described in positive and negative expression (5 for type-1- and 4 for type-2-alliances). Positive type 1: wanting to reach the patient’s goals, understanding, warmth/receptiveness, hopefulness, recognition of progress. Positive type 2: establishment of „we“-bond, acceptance of patient‘s autonomy, reference to past problems they have overcome together, supportiveness. | Behaviors and statements serve as indicators („signs“) of a subtype. Signs are counted and their intensity is rated on a 5-point-likert-scale. | O | Interrater reliabilty (correlation of agreement): r = .47-.83 (dependent on positive or negative indicators and timepoint in therapy) |
|  | Helping Alliance Rating Method (HAr; Morgan et al., 1982) | Based on Luborsky (1976) | 6 indicators for type-1- and 4 indicators for type-2-alliance are described with patient-behaviors in 1-2 sentences.  Accordingly, 5 therapist-behaviors, that are characteristic for type-1- and type-2-alliances respectively, are described. | 10 indicators for TA;  10-point-likert-scale | O | Interrater reliability = .75-.88  α = .96 |
|  | Helping Alliance Questionnaire 1 (HAQ-1; Eich et al., 2018) | Based on Luborsky (1976) | 2 factors: relation to therapist/patient (Type-1-Alliance) and satisfaction with outcome (Type-2-Alliance). | 11 items;  6-point-likert-scale | P, T | α = .63-.89 (dependent on subscale and perspective) |
|  | Helping Alliance Question-naire 2 (HAQ-2; Luborsky et al., 1996) | Based on Luborsky (1976) and Bordin (1979) | 2 factors: „Positive Therapeutic Alliance“ and „Negative Therapeutic Alliance“ which were discarded due to their high intercorrelation. | 19 items  6-point-likert-scale | P, T | α = .90-.93 (dependent on subscale and perspective) |
| Helping Alliance Scales (HAS) | Priebe & Gruyters, 1993 | No theoretical foundation. Focus on TA in psychiatric community care. |  | 5 questions;  10-cm-long Visual Analog Scales  The last question can only be answered with „yes“ or „no“. | P | NA |
| Individual Therapy Alliance Scale – Revised – Short Form (ITASr-SF) | Karam et al., 2015; a complete copy of the items can be found in Pinsof et al., 2008. There are versions for couples- and family-PT. | Based on Bordin (1979) an on Pinsof & Catherall (1986). Pinsof and collegues (Pinsof & Catherall, 1986; Pinsof et al., 2008) view TA from a systemic point of view. There are 2 domains of TA in their model. The Content domain characterizes TA with Bordin’s factors. The Interpersonal System domain speceifies, who is involved (e.g. patient-therapist, patient’s partner-therapist, ...). | Items can be sorted into a 3x4-matrix that reflects Bordin’s 3 factors and 4 relationship constellations (patient-therapist, therapist and one relevant other of the patient, therapist and friend group of the patient, patient and his*her friend group). | 15 items;  7-point-likert-scale | P | α = .25-.75 (dependent on subscale) |
| Interview Rating Scale (IRS) | Anderson & Anderson, 1962 | Based on Fiedler’s (1950) survey, their own survey of patients and therapists and the lecture of case studies. Fiedler (1950) asked therapists to rate 119 sentences that described TA. He found that there was a similar concept of the ideal TA in different therapy schools. | 18 items related to patient-behaviors, 32 items related to therapist-behaviors. | 50 items;  5-point-likert-scale | P, T | NA |
| Kim Alliance Scale (KAS) | Kim et al., 2001 | Literature search for conceptualizations and operationalizations of TA (deductive process);  qualitative survey of medical staff about key words for TA (inductive process). | KAS und KAS-R are composed of 4 scales: „Collaboration“, „Communication“, „Integration“ and „Empowerment“. | 30 items;  4-point-likert-scale | P | α = .94 (.71-.87)*** |
| Kim Alliance Scale – revised (KAS-R) | Kim et al., 2008 | Revised short version of the KAS (Kim et al., 2001). | See above | 16 items;  4-point-likert-scale | P | α = .89 (.75-.80)*** |
| Menninger Scales to Asses the Therapeutic Alliance | Allen et al., 1984 | Based on Frieswyck et al. (1984): They differentiate between the patient‘s active participation, the patient’s relationship experiences and therapeutic techniques. | Core-scale for measuring TA: „Collaboration-Scale“; additionally 4 scales for measuring patient-related mediating variables of TA: „Trust“, „Sense of accceptance“, „Optimism“ and „Expression of affect“. | There is one 5-point-scale each for the Collaboration scale and the mediating variables. Each level is described in detail. Sublevels can also be assigned. | T, O | Interrater reliability with Spearman Brown corrected reliability coefficients = .60-.79 (dependent on subscale) |
| Psycho-therapy Relationship Question-naire (PRQ) | Bradley et al., 2005 | Literature search for phenomenoms referred to as „transference“ and „working allilance“ | 5 factors: „angry/entitled“, „anxious/preoccupied“, „avoidant/ counterdependent“, „secure/engaged“ and „sexualized“. | 90 items;  5-point-likert-scale | T | α = .84-.94 (dependent on subscale) |
| Psycho-therapy Status Report (PSR) | Frank & Gunder-son, 1990 | No theoretical foundation. | 6 scales: 1. „stable, active, and collaborative participation in the treatment process“; 2. „full and spontaneous sharing of relevant material with the therapist and responsiveness to the therapist's interventions“; 3. „express valuing of the psychotherapy and optimism about its potential usefulness“; 4. „maintenance of a sustained interest in understanding the illness and its influence on self and others, in problem solving, and in pursuing therapeutic goals“; 5. „presence of a generally clear and realistic picture of the therapist and the therapeutic relationship, and amenability to confrontation or interpretation of transference or other distortions of that relationship“; 6. „affective involvement in the treatment and relatedness to the therapist“. | Each scale has 5 levels defined by clinical descriptors. | T | Retest reliability (3 month interval): r = .89  α = .72 |
| Real Relation-ship Inventory (RRI) | RRI-T: Gelso et al., 2005;  RRI-C: Kelley et al., 2010 | Based on Gelso & Carter (1985; 1994). According to Greenson (1967), TA is made up of 3 components: transference, working alliance and the real relationship, which is later picked up by Gelso and Carter (Gelso & Carter, 1985; Gelso & Carter, 1994; Gelso et al., 2005). The real relationship refers to the personal aspects aside from the professional relationship (Gelso et al., 2005; Hougaard, 1994). In contrast to transference, the real relationship is grounded in reality and contains 2 elements: genuiness and the realisitc perception of the other (Gelso & Carter, 1994; Gelso et al., 2005). | 2 scales: „Genuineness“ and „Realism“. | 24 items;  5-point-likert-scale | P, T | T: α = .72-.89 (dependent on subscale and sample)  C: α = .86-.91 (dependent on subscale and sample);  retest reliability (2-3 week-interval): r = .87 (.84-.88)*** |
| Relational Depth Frequency Scale (RDFS) | Di Malta et al., 2019 | Based on Mearns und Cooper (2005, 2018): They focus on relational depth, which is defined as a state of profound contact and engagement between people (Mearns & Cooper, 2018, Preface S. xvii). | Unifactorial structure of relational depth. | 6 items;  5-point-likert-scale | P, T | α = .85-.93 (dependent on perspective) |
| Relation-ship Inventory (RI) | Barrett-Lennard, 1962 | Rogers (1957) client-centered concept of TA, in which necessary and sufficient conditions for therapeutic personality change are formulated. | 5 scales: „Level of regard“, „Empathetic understanding“, „Congruence“, „Unconditionality of regard“ and „Willingness to be known“. | 92 items;  6-point-scale | P | Split-Half-reliability (Spearman-Brown) = .82-.96 (dependent on subscale and perspective) |
| Rupture Resolution Rating System (3RS) | Eubanks et al., 2018 | Based on Safran & Muran (2000) | Patient-behaviors serve as indicators for conflicts („rupture markers“), differentiation between conflicts due to confrontation and to withdrawal;  Therapist-behaviors serve as indicators for resolution strategies. | 14 rupture markers and 10 resolution strategies are described.  A session in divided into 5-min.-segments. The global extent of ruptures and resolutions in assesed on a 5-point-likert-scale. The corresponding markers are counted and rated according to their significance. | O | Inrerrater-reliability (ICC) = .85-.98 |
| Rupture Resolution Question-naire (RRQ) | Safran et al. (2008)* | Based on Safran & Muran (2000); Safran et al. (2002). |  | 18 items;  5-point-likert-scale | P | α = .87 |
| Session Rating Scale (SRS) | Murphy et al., 2020 | 3 items are based on Bordin (1979), the 4th item measures the overall patient-satisfaction. | The 4 items are named „Relationship“, „Goals and Topics“, „Approach or Method“ and „Overall“. | 4 items;  10-cm-long Visual Analog Scales | P | Reported retest reliabilities: r = .54-.88  Reported α = .69-.97 |
| Scale To Asses The Therapeutic Relationship (STAR) | McGuire-Snieckus et al., 2007;  German version: Gairing et al., 2011 | Qualitative survey of clinical staff of psychiatric community care. | Patient-version (STAR-P): 3 subscales „Positive collaboration“, „Positive clinician input“ und „Non-supportive clinincian input“.  Clinician-Version (STAR-C): The last subscale is substituted by „clinician emotional difficulties“. | 12 items;  5-point-likert-scale | P, T | English version: α = .71-.98 (dependent on subscale and perspective); retest-reliability (2 week interval): r= .68-.76 (dependent on perspective)  German version: α = .28-.89 (dependent on subscale and perspective); retest-reliability (after approx. 29 days): r = .54-.68 (dependent on perspetive) |
| Skala Therapeu-tische Allianz – Revised (STA-R) | Brockmann et al., 2011 | Adaptation of the unpublished Combined Alliance Scale (CAS) by Hatcher & Shannon (2005**), which was mainly based on a factor analysis of the WAI, HAQ and CALPAS (Hatcher & Barends, 1996). | 4 scales: „Confident Collaboration, „Bond“, „Patient Fear“ and „Therapist Interference“. | 17 items;  5-point-likert-scale | P | α = .62-.87 (dependent on subscale) |
| Therapeutic Alliance Rating System (TARS) | Marmar et al., 1986; Marziali, 1984; Marziali et al., 1981 (no access to items) | Based on psychodynamic conceptualizations and on Bordin (1979), focus on affective aspects (Ardito &Rabellino, 2011; Elvins & Green, 2008). Integration of items from HAcs (Luborsky, 1983), VPPS (Strauß, 1991) and VTAS (Hartley & Strupp, 1983). | 4 scales: „Therapist Positive Contribution“, „Therapist Negative Contribution“, „Patient Positive Contribution“, „Patient Negative Contribution“ (Eaton et al., 1988) | 42 items;  6-point-scale | P, T, O | Adequate internal consistencies (Elvins & Green, 2008) |
| Therapeu-tic Bond Scales (TBS) | Saunders et al., 1989 | Based on Orlinsky & Howard and their TSR (1967, 1975, 1978, 1986) Orlinsky and Howard (1975, 1978; 1986) synthesize results from empirical research and propose 3 dimensions of TA: 1. Role-investment or working alliance, 2. Empathetic resonance 3. Mutual affirmation (Elvins & Green, 2008; Saunders et al., 1989). | 3 scales: „Working Alliance Scale“, „Empathetic Resonance Scale“ and „Mutual Affirmation Scale“. | 50 items;  Scale from 0 to 20 | P | α = .62 (.72-.87)*** |
| Vanderbilt Psycho-therapy Process-Scale (VPPS) | VPPS: Strauß, 1991 | Based on the TSR (Orlinsky & Howard, 1967, 1975) | 39 items related to patient-behaviors and -attitudes; 36 items that are related to therapists.  3 questions concerning the global impression of the session and the patient’s state | 75 +3 items;  5-point-scale; sublevels can be assigned | O | α = .96-.82 (dependent on subscale)  Interrater-reliability = .94-.79  (Elvins & Green, 2008) |
| Vanderbilt Thera-peutic Alliance Scale (VTAS) | Hartley & Strupp, 1983 (no access to items) | The VTAS derived from the VPPS, in order to specifically measure TA and integrates several theories: Bordin (1979), Greenson (1965), Luborsky (1976) and Langs (1973) emphasis of an open and lively storytelling of patients (Shelef & Diamond, 2008; Elvins & Green, 2008; Ardito & Rabellino, 2011). | 3 scales: „Therapist Contributions“, „Patient Contributions“, „Patient-Therapist-Interaction“ (Shelef & Diamond, 2008) | 44 items;  6-point-scale | O | Solid interrater-reliability and internal consistency (Elvins & Green, 2008) |
| Vanderbilt Therapeutic Alliance Scale – Revised Short Form (VTAS-R-S) | VTAS-R-S: Shelef & Diamond, 2008 | Focus on Bordin (1979) | 3 items referring to patient-behaviors, 2 items referring to interactional aspects | 5 items;  6-point-likert-scale | O | Interrater reliability (ICC) = .72-.87 (dependent on item)  α = .90-.91 (dependent on who is rated by observers) |
| Working Alliance Inventory (WAI) | Horvath & Greenberg, 1989 | Bordin‘s (1979) conceptualization; for further versions see wai.profhorvath.com | 3 scales: Tasks, Goals and Bond | 36 items;  5-point-likert-scale | P, T, O | Overall α = .87-.93 (dependent on perspective)  Subscales α = .68-.92 (dependent on perspective) |
|  | WAI-T: Hatcher et al., 2020 (full version from wai.profhorvath.com) |  | 3 scales: Tasks, Goals and Bond | 36 items;  7-point-likert-scale | T | α = .87 (Horvath & Green, 1989) |
|  | Brief Alliance Inventory (BAI; Mallinckrodt & Tekie, 2015) |  | 3 scales: Tasks, Goals and Bond | 16 items;  5-point-likert-scale | P | α = .89-.93 (dependent on subscale) |
|  | WAI-S: Hukkel-berg & Ogden, 2017 |  | 3 scales: Tasks, Goals and Bond | 12 items;  7-point-likert-scale | P, T | Omega = .96 (.87-.92) |
|  | WAI-S-R: Falkenström et al., 2015a |  | 2 scales: Tasks/Goals and Bond | 12 items; Falkenström et al. (2015a) report the implementation of a 5-as well as a 7-point-likert-scale. | P, T | NA |
|  | Session Alliance Inventory (SAI; Falkenström et al., 2015b) |  | 2 scales: Tasks/Goals and Bond | 6 items;  5-point-likert-scale | P | Omega = .89-.94 (dependent on sample) |
|  | S-WAI-O: Berk et al., 2013 |  | 2 scales: Tasks and Bond | 12 items;  7-point-likert-scale | O | Interrater reliability (ICC) = .856 |
| **Online-Therapy** |  |  |  |  |  |  |
| Working Alliance Inventory for guided Internet Interventions (WAI-I) | Gómez Penedo et al., 2020 | Adaptation of the WAI for guided internet interventions: Patients independently work through units, but have regular contacts with a therapist. | 2 scales: Tasks/Goals and Bond | 12 items;  5-point-likert-scale | P | α = .93 (.89-.93)*** |
| Working Alliance Inventory for Online Interventions – Short Form (WAI-TECH-SF) | Herrero et al., 2020 | Adaptation of the WAI-S for internet-based interventions. TA between the patient and an online-program is measured. An additional accompaniment by a therapist can be provided. | Unifactorial structure | 12 items;  7-point-likert-scale | P | α = .97 |

* Unpublished measure by Safran et al. (1996); retrieved from Safran et al. (2008)

** Since the CAS remained unpublished, this source was not accessible for further investigation.

Notes. 4PAS = 4-Point-Alliance-Scale; ABAQ = Attachment Based Alliance Questionnaire; AiA = Alliance in Action; ANS = Alliance Negotiation Scale; ARM = Agnew-Davies Relationship Measure; ARM-5 = 5-Item Agnew-Davies-Relationship Measure; ARM-12 = 12-Item Agnew-Davies Relationship Measure; AROS = Alliance and Rupture Observation Scale; BAI = Brief Alliance Inventory; BQTA = Brief Questionnaire on Therapeutic Alliance; Brief-CALPAS = 5-Item California Psychotherapy Alliance Scales; CALPAS = California Psychotherapy Alliance Scales; CAS = Combined Alliance Scale; CEI = Counselor Evaluation Inventory; CRF = Counselor Rating Form; HAcs = Helping Alliance Counting Signs; HAQ-1 = Helping Alliance Questionnaire 1; HAQ-2 = Helping Alliance Questionnaire 2; HAr = Helping Alliance Rating Method; HAS = Helping Alliance Scale; IRS = Interview Rating Scale; ITASr-SF = Individual Therapy Alliance Scale revised – Short Form; KAS = Kim Alliance Scale; KAS-R = Kim Alliance Scale – Revised; O = Observer; OP = Online Program; P = Patient; PRQ = Psychotherapy Relationship Questionnaire; PSR = Psychotherapy Status Report; RDFS = Relational Depth Frequency Scale; RI = Relationship Inventory; RRI-C = Real Relationship Inventory – Client Version; RRI-T = Real Relationship Inventory – Therapist Version; RRQ = Real Relationship Questionnaire; 3RS = Rupture Resolution Rating System; SAI = Session Alliance Inventory; SRS = Session Rating Scale; STAR-P = Scale to Asses Therapeutic Relationship – Patient Version; STAR-C = Scale to Asses Therapeutic Relationship – Clinician Version; STA-R = Skala Therapeutische Allianz – Revised; S-WAI-O = Segmented Working Alliance Inventory – Observer Version; T = Therapist; TA = Therapeutic Alliance; TBS = Therapeutic Bond Scales; TSR = Therapy Session Report; VTAS-S-R = Vanderbilt Therapeutic Alliance Scale – Short Form – Revised; VPPS = Vanderbilt Psychotherapy Process Scale; WAI = Working Alliance Inventory; WAI-I = Working Alliance Inventory for guided Internet Interventions; WAI-S = Working Alliance Inventory – Short Form; WAI-S-R = Working Alliance Inventory – Short Form – Revised; WAI-T = Working Alliance Inventory – Therapist Version; WAI-TECH-SF = Working Alliance Inventory for Online Interventions – Short Form.

***When reliability estimates are provided in this form (α = .xx (.xy-.xz)), the first estimate refers to the estimate for the entire scale, whereas the estimates inside the parentheses refer to the estimates for the subscales.

**Table S2: Sources of Included Articles in the Context of Face-to-Face-Psychotherapy**

| Identification of studies via systematic search | Identification of studies via other methods | |
| --- | --- | --- |
| Systematic search | Reference lists | Retrieved from past reviews |
| 1. 3RS (Eubanks et al., 2018) 2. 4PAS (Misdrahi et al., 2009) 3. ABAQ (Johnson et al., 2018) 4. AiA (Owen et al., 2013) 5. ANS (Doran et al., 2012) 6. AROS (Holmqvist Larsson et al., 2019) 7. BAI (Mallinckrodt & Tekie, 2015) 8. BQTA (Widschwendter et al., 2016) 9. Brief CALPAS (Daniels & Wearden, 2011) 10. ITASr-SF (Karam et al., 2015; full list of items provided in Pinsof et al., 2008) 11. RDFS (Di Malta et al., 2019) 12. RRI-C (Kelley et al., 2010) 13. SAI (Falkenström et al., 2015b) 14. SRS (Murphy et al., 2020) 15. STAR (Deutsche Version; Gairing et al., 2011) 16. STA-R (Brockmann et al., 2011) 17. WAI-S (Hukkelberg & Ogden, 2017) 18. WAI-S-R (Falkenström et al., 2015a) 19. WAI-T (Hatcher et al., 2020) | 1. ARM-5; ARM-12 (Cahill et al., 2012) 2. IRS (Anderson & Anderson, 1962) 3. KAS-R (Kim et al., 2008) 4. PRQ (Bradley et al., 2005) 5. RRI-T (Gelso et al., 2005) 6. RRQ (Safran et al., 1996; entnommen aus und zitiert nach Safran et al., 2008) 7. S-WAI-O (Berk et al., 2013) 8. VTAS-S-R (Shelef & Diamond, 2008) | 1. ARM (Agnew-Davies et al., 1998) 2. CALPAS (Gaston & Marmar, 1993*) 3. CEI (Linden et al., 1965) 4. CRF (Barak & LaCrosse, 1975) 5. HAS (Priebe & Gruyters, 1993) 6. HAcs (Luborsky et al., 1983) 7. HAr (Morgan et al., 1982) 8. HAQ-1 (Eich et al., 2018**) 9. HAQ-2 (Luborsky et al., 1996) 10. KAS (Kim et al., 2001) 11. Menninger (Allen et al., 1984) 12. PSR (Frank & Gunderson, 1990) 13. RI (Barrett-Lennard, 1962) 14. STAR (McGuire-Snieckus et al., 2007) 15. TARS (Marziali, 1984; Marziali et al., 1981) 16. TBS (Saunders et al., 1989) 17. VPPS (Strauß, 1991) 18. VTAS (Hartley & Strupp, 1983) 19. WAI (Horvath & Greenberg, 1989) |

**Notes.** 3RS = Rupture Resolution Rating System; 4PAS = 4-Point-Alliance-Scale; ABAQ = Attachment Based Alliance Questionnaire; AiA = Alliance in Action; ANS = Alliance Negotiation Scale; ARM = Agnew-Davies Relationship Measure; ARM-5 = 5-Item Agnew-Davies-Relationship Measure; ARM-12 = 12-Item Agnew-Davies Relationship Measure; AROS = Alliance and Rupture Observation Scale; BAI = Brief Alliance Inventory; BQTA = Brief Questionnaire on Therapeutic Alliance; Brief-CALPAS = 5-Item California Psychotherapy Alliance Scales; CALPAS = California Psychotherapy Alliance Scales; CEI = Counselor Evaluation Inventory; CRF = Counselor Rating Form; HAS = Helping Alliance Scale; HAcs = Helping Alliance Counting Signs; HAr = Helping Alliance Rating Method; HAQ-1 = Helping Alliance Questionnaire 1; HAQ-2 = Helping Alliance Questionnaire 2; IRS = Interview Rating Scale; ITASr-SF = Individual Therapy Alliance Scale revised – Short Form; KAS = Kim Alliance Scale; KAS-R = Kim Alliance Scale – Revised; Menninger = Menninger Alliance Scales; PRQ = Psychotherapy Relationship Questionnaire; PSR = Psychotherapy Status Report; RDFS = Relational Depth Frequency Scale; RI = Relationship Inventory; RRI-C = Real Relationship Inventory – Client Version; RRI-T = Real Relationship Inventory – Therapist Version; RRQ = Real Relationship Questionnaire; SAI = Session Alliance Inventory; SRS = Session Rating Scale; STAR = Scale to Asses Therapeutic Relationship; STA-R = Skala Therapeutische Allianz – Revised; S-WAI-O = Segmented Working Alliance Inventory – Observer Version; TARS = Therapeutic Alliance Rating Scales; TBS = Therapeutic Bond Scales; VPPS = Vanderbilt Psychotherapy Process Scale; VTAS = Vanderbilt Therapeutic Alliance Scale; VTAS-S-R = Vanderbilt Therapeutic Alliance Scale – Short Form – Revised; WAI = Working Alliance Inventory; WAI-T = Working Alliance Inventory – Therapist Version

*This source was found by manual search for a complete list of the CALPAS-Items, after the instrument had been introduced in the reviews.

**The measurement types of the Penn Scales (Rating method, Counting Signs, Questionnaire) were broadly presented in the reviews. In search of specific corresponding sources for the items of the Helping Alliance Questionnaires (HAQ), the cited papers above were obtained. This explains the publishing year of 2018 for the HAQ-1.

**Table S3: Inter-Rater-Reliabilities in Qualitative Content Analysis**

| **Instrument** | **Number of overlapping subcategories** | **Total number of used subcategories** | **Percentage of agreement** |
| --- | --- | --- | --- |
| 3RS (Eubanks et al., 2018) | 9 | 15 | 60,00 |
| 4PAS (Misdrahi et al., 2009) | 8 | 10 | 80,00 |
| ABAQ (Johnson et al., 2018) | 8 | 13 | 61,54 |
| AiA (Owen et al., 2013) | 6 | 10 | 60,00 |
| ANS (Doran et al., 2012) | 8 | 11 | 72,73 |
| ARM (Agnew-Davies et al., 1998) | 28 | 34 | 82,35 |
| AROS (Holmqvist Larsson et al., 2018) | 25 | 32 | 78,13 |
| BQTA (Widschwendter et al., 2016) | 5 | 9 | 55,56 |
| Brief CALPAS (Muran et al., 1995) | 6 | 8 | 75,00 |
| CALPAS (Gaston & Marmar, 1993) | 25 | 34 | 73,53 |
| CEI (Linden et al., 1965) | 17 | 20 | 85,00 |
| CRF (Barak & LaCrosse, 1975) | 18 | 29 | 62,07 |
| HAcs (Luborsky et al., 1983) | 28 | 33 | 84,85 |
| HAQ-1 (Eich et al,. 2018) | 11 | 14 | 78,57 |
| HAQ-2 (Luborsky et al., 1996) | 21 | 26 | 80,77 |
| HAr (Morgan et al., 1982) | 18 | 23 | 78,26 |
| HAS (Priebe & Gruyters, 1993) | 6 | 8 | 75,00 |
| IRS (Anderson & Anderson, 1962) | 31 | 42 | 73,81 |
| ITASr-SF (Pinsof et al., 2008) | 7 | 8 | 87,50 |
| KAS (Kim, 2001) | 9 | 9 | 100,00 |
| KAS-R (Kim et al., 2008) | 16 | 17 | 94,12 |
| Menninger (Allen et al., 1984) | 30 | 31 | 96,77 |
| PRQ (Bradley et al., 2005) | 22 | 26 | 84,62 |
| PSR (Frank & Gunderson, 1990) | 10 | 13 | 76,92 |
| RDFS (Di Malta et al., 2019) | 7 | 8 | 87,50 |
| RI (Barrett-Lennard 1962) | 26 | 28 | 92,86 |
| RRI-C (Kelley et al., 2010) | 20 | 23 | 86,96 |
| RRI-T (Gelso et al., 2005) | 16 | 20 | 80,00 |
| RRQ (Safran et al., 1996) | 7 | 11 | 63,64 |
| SRS (Murphy et al., 2020) | 12 | 16 | 75,00 |
| STAR (German; Gairing et al., 2011) | 17 | 18 | 94,44 |
| STAR (English; McGuire-Snieckus et al., 2007) | 21 | 23 | 91,30 |
| STA-R P (Brockmann et al., 2011) | 11 | 19 | 57,89 |
| TBS (Saunders et al., 1989) | 20 | 28 | 71,43 |
| VPPS (Strauß, 1991) | 34 | 43 | 79,07 |
| VTAS-S-R (Shelef & Diamond, 2008) | 5 | 7 | 71,43 |
| WAI (Horvath & Greenberg, 1989) | 25 | 32 | 78,13 |
| WAI-T (profhorvath.com) | 20 | 28 | 71,43 |
| <Total> | 613 | 779 | 78,69 |

**Notes.** 3RS = Rupture Resolution Rating System; 4PAS = 4-Point-Alliance-Scale; ABAQ = Attachment Based Alliance Questionnaire; AiA = Alliance in Action; ANS = Alliance Negotiation Scale; ARM = Agnew-Davies Relationship Measure; AROS = Alliance and Rupture Observation Scale; BQTA = Brief Questionnaire on Therapeutic Alliance; Brief-CALPAS = 5-Item California Psychotherapy Alliance Scales; CALPAS = California Psychotherapy Alliance Scales; CEI = Counselor Evaluation Inventory; CRF = Counselor Rating Form; HAcs = Helping Alliance Counting Signs; HAQ-1 = Helping Alliance Questionnaire 1; HAQ-2 = Helping Alliance Questionnaire 2; HAr = Helping Alliance Rating Method; HAS = Helping Alliance Scale; IRS = Interview Rating Scale; ITASr-SF = Individual Therapy Alliance Scale revised – Short Form; KAS = Kim Alliance Scale; KAS-R = Kim Alliance Scale – Revised; Menninger = Menninger Alliance Scales; PRQ = Psychotherapy Relationship Questionnaire; PSR = Psychotherapy Status Report; RDFS = Relational Depth Frequency Scale; RI = Relationship Inventory; RRI-C = Real Relationship Inventory – Client Version; RRI-T = Real Relationship Inventory – Therapist Version; RRQ = Real Relationship Questionnaire; SRS = Session Rating Scale; STAR = Scale to Asses Therapeutic Relationship; STA-R-P = Skala Therapeutische Allianz – Revised – Patient Version; TBS = Therapeutic Bond Scales; VPPS = Vanderbilt Psychotherapy Process Scale; VTAS-S-R = Vanderbilt Therapeutic Alliance Scale – Short Form – Revised; WAI = Working Alliance Inventory; WAI-T = Working Alliance Inventory – Therapist Version.

**Table S4: Results of Qualitative Content Analysis**

| **Category/subcategory** | **Number of items in subcategory** | **Number of items per instrument in subcategory** | **Examples of items** |
| --- | --- | --- | --- |
| **Neutral terms** |  |  |  |
| Agreement upon problem-causes, goals, methods | 53 | 1 4PAS; 4 ARM; 1 AROS; 2 BQTA; 2 Brief-CALPAS; 9 CALPAS; 1 HAcs; 2 HAQ-1; 2 HAQ-2; 1 HAr; 2 KAS-R; 3 VTAS-R; 1 SRS; 4 STAR-E; 4 STAR-D; 2 TBS; 5 WAI; 7 WAI-T | My patient and I are working on common goals.  I believe we have similar ideas about the nature of my problems. |
| Experienced quality of relationship – other | 26 | 1 ARM, 2 BQTA; 1 HAQ-2; 2 HAr; 1 KAS; 2 RDFS; 3 RRI-C; 3 RRI-T; 2 RRQ; 3 STAR-E; 3 STAR-D; 1 TBS; 1 WAI; 1 WAI-T | We were deeply connected to one another. |
| Collaboration as a team | 24 | 1 4PAS; 1 IRS; 2 ARM; 1 AROS; 1 Brief CALPAS; 2 CALPAS; 4 HAcs; 2 HAQ-1; 2 HAQ-2; 2 HAr; 1 WAI; 2 WAI-T | I feel that my doctor and I work together as a team. |
| Other | 20 | 1 3RS; 1 IRS; 3 AROS; 2 HAr; 5 KAS; 2 KAS-R; 1 SRS; 1 STA-R; 3 VPPS |  |
| Disagreement | 16 | 1 ANS; 2 AROS; 3 CALPAS; 1 HAcs; 2 ITASr-SF; 2 SRS; 2 WAI; 3 WAI-T | The patient has different conceptions about the etiology of his problems.  How much did you disagree with your therapist about what issues were most important to work on during this session? |
| Real relationship next to TA | 11 | 3 RRI-C; 7 RRI-T; 1 TBS | I feel there is a “real” relationship between us aside from the professional relationship.  My therapist liked the real me. |
| Relationship of patient’s close ones with therapist | 10 | 10 ITASr-SF | Some of the people who are important to me would not be pleased with what I am doing in this therapy.  The people who are important to me would feel accepted by the therapist. |
| Mutual understanding | 10 | 2 ARM; 3 AROS; 1 RDFS; 1 RRI-C; 1 RRI-T; 1 STA-R; 1 WAI | The relationship between my therapist and me was strengthened by our understanding of one another. |
| Bad collaboration | 8 | 2 ARM; 1 AROS; 4 HAcs; 1 Menninger | Very strong evidence against collaboration in current activity. |
| Mutual trust | 7 | 1 AROS; 2 STAR-E; 2 STAR-D; 1 WAI; 1 WAI-T | My clinician and I share a trusting relationship. |
| Deep, meaningful conversation | 4 | 1 AROS; 2 HAQ-2; 1 RDFS | Extremely intimate/emotionally challenging details or problems are disclosed. |
| Time-use, presence in here and now | 4 | 2 RDFS; 1 WAI; 1 WAI-T | We were immersed in the present moment. |
| Conflicts and conflict resolution | 4 | 1 3RS; 1 ANS; 2 RRQ | Therapist responds to a rupture by changing tasks or goals. |
| Mutual non-understanding | 2 | 1 AROS; 1 WAI | Participants do not in any way understand the other’s opinions. |
| Realistic view of relationship | 1 | 1 RRI-C | My therapist and I had a realistic perception of our relationship. |
| Non-useful topics | 1 | 1 HAQ-2 | The therapist and I sometimes have unprofitable exchanges. |
| Lack of mutual trust | 1 | 1 AROS | Demonstration of complete lack of confidence/trust in the other. |
| **Patient terms** |  |  |  |
| Self-disclosure, authenticity | 55 | 3 IRS; 1 ANS; 3 ARM; 7 CALPAS; 1 HAr; 1 KAS; 2 Menninger; 2 PRQ; 3 PSR; 5 RRI-C; 4 RRI-T; 4 RRQ; 3 STAR-E; 3 STAR-D; 3 TBS; 8 VPPS | Patient frequently volunteers relevant, personal material and is responsive on a meaningful level, giving feedback and elaborating on feelings and problems. |
| Active participation, motivation, compliance | 52 | 1 3RS; 4 ARM; 4 BQTA; 14 CALPAS; 1 HAcs; 1 HAQ-1; 2 HAQ-2; 1 HAr; 1 KAS; 1 KAS-R; 2 Menninger; 2 PRQ; 2 PSR; 8 TBS; 7 VPPS; 1 WAI | The patient likes to come to the appointments and attends reliably.  I am committed to reach the goals.  Patient works actively with the therapist’s comments.  The patient is making optimal use of the treatment as a resource for constructive change, participating actively in the requisite treatment tasks. He or she spontaneousely brings into the treatment all the significant issues and openly conveys the associated information and feelings. |
| Trust and secure attachment towards therapist | 52 | 2 4PAS; 1 ABAQ; 3 IRS; 2 ANS; 7 ARM; 1 AROS; 2 CALPAS; 2 CEI; 3 HAQ-1; 1 HAQ-2; 4 Menninger; 3 PRQ; 1 RI; 3 RRI-C; 1 RRI-T; 7 RRQ; 1 STAR-E; 1 STAR-D; 1 STA-R; 2 TBS; 2 VPPS; 2 WAI | I felt at ease with the counselor.  I feel I can depend on my therapist.  Feels nurtured by the therapist.  I was able to be myself with my therapist.  How free were you to discuss personal matters that you are ordinarily ashamed or afraid of to reveal? |
| Learning, remission | 41 | 2 4PAS; 2 IRS; 4 CALPAS; 1 CEI; 2 HAcs; 7 HAQ-1; 5 HAr; 2 Menninger; 5 RRQ; 2 STA-R; 4 TBS; 2 VPPS; 2 WAI; 1 WAI-T | I have a better understanding of the symptoms of my illness. |
| Avoidance/ withdrawal/ pretending/ trivializing | 34 | 6 3RS; 2 ABAQ; 1 ANS; 2 ARM; 1 AROS; 2 CALPAS; 3 Menninger; 3 PRQ; 1 PSR; 4 RRI-C; 1 RRI-T; 1 RRQ; 2 STA-R; 1 TBS; 4 VPPS | I keep some important things to myself, not sharing them with my therapist.  Patient withdraws from the therapist and/or work of therapy by denying a feeling state that is manifestly evident, or denying the importance of interpersonal relationships or events that seem important and relevant to the work of therapy. |
| Vigilance, suspiciousness, distrust | 31 | 5 ABAQ; 3 IRS; 1 ANS; 3 ARM; 1 CEI; 1 HAQ-2; 5 Menninger; 1 PRQ; 1 RRI-C; 1 VTAS-S-R; 3 STA-R; 2 TBS; 2 VPPS; 1 WAI; 1 WAI-T | I distrusted the counselor.  The patient may believe that he or she needs to be careful about behavoir, both in and out of therapy, lest he or she elicit the therapist’s dislike. |
| Confidence in therapist/hopefulness towards therapy | 30 | 1 IRS; 4 ARM; 1 BQTA; 6 CALPAS; 1 HAcs; 3 HAQ-1; 1 HAr; 1 HAS; 1 Menninger; 1 PRQ; 1 PSR; 2 STA-R; 3 TBS; 1 VPPS; 2 WAI, 1 WAI-T | I have confidence in my therapist and his/her techniques. |
| Passiveness, lets therrapist take responsibility, refusing | 27 | 1 3RS; 2 ARM; 1 AROS; 2 BQTA; 1 CALPAS; 1 HAcs; 2 Menninger; 4 PRQ; 2 PSR; 1 VTAS-S-R; 1 STA-R; 2 TBS; 6 VPPS; 1 WAI-T | Patient rejects or dismisses the therapist’s intervention.  Der Patient verließ sich bei der Lösung seiner Probleme auf den Therapeuten. |
| Feels disliked/unaccepted | 24 | 3 ABAQ; 1 IRS; 1 ARM; 2 HAcs; 1 HAS; 1 ITASr-SF; 1 KAS-R; 2 Menninger; 1 PRQ; 8 RI; 1 SRS; 1 TBS; 1 VPPS | He feels that I am dull and un-interesting.  I do not feel accepted by the therapist. |
| Feels liked/accepted | 24 | 2 IRS; 1 CALPAS; 1 CEI; 1 HAcs; 1 HAQ-2; 2 HAr; 1 KAS-R; 2 Menninger; 5 RI; 1 RRI-C; 1 SRS; 2 STA-R; 2 TBS; 2 WAI | He likes seeing me. |
| Dependence/ lack of autonomy | 24 | 3 ABAQ; 1 HAcs; 16 PRQ; 4 VPPS | Is afraid of contradicting or disagreeing with the therapist; has trouble asserting own needs or viewpoint.  Is afraid of being abandoned by the therapist.  Submissive. |
| Conflict & critique | 22 | 4 3RS; 2 ARM; 2 AROS; 1 Menninger; 12 PRQ; 1 VPPS | Repeatedly tests or fails to respect the boundaries of the therapeutic relationship.  Voices concerns that the therapist is not doing enough to help. |
| No confidence in therapist/no hopefulness towards therapy | 21 | 2 3RS, 1 ABAQ; 1 AROS; 1 CALPAS; 2 HAcs; 1 HAQ-2; 4 Menninger; 1 PRQ; 1 PSR; 2 TBS; 2 VPPS; 2 WAI; 1 WAI-T | Worries that the therapist cannot help him/her. |
| Feels understood/seen | 19 | 1 4PAS; 2 BQTA; 1 CALPAS; 1 HAcs; 2 HAQ-1; 1 HAQ-2; 1 HAr; 1 HAS; 3 RI; 1 RRI-C; 1 VTAS-S-R; 1 SRS; 1 STAR-E; 1 STAR-D; 1 WAI | I feel my doctor really understands me. |
| Dissatisfaction with therapy | 16 | 1 IRS; 2 ARM; 2 AROS; 2 CALPAS; 2 HAcs; 1 HAQ-2; 2 PSR; 1 TBS; 1 VPPS; 1 WAI; 1 WAI-T | My client is critical or disappointed in me.  Patient is consistently negative regarding the usefulness of therapy, and expressed feelings are of getting nowhere in treatment. |
| Satisfaction with therapy | 13 | 1 ABAQ; 1 IRS; 1 BQTA; 2 Brief-CALPAS; 1 CALPAS; 3 CEI; 1 HAr; 1 PRQ; 2 TBS | I felt satisfied as a result of my talks with the counselor. |
| Doesn’t feel understood/seen | 13 | 1 ARM; 1 AROS; 1 HAcs; 1 HAQ-2; 1 ITASr-SF; 1 Menninger; 3 RI; 1 RRI-C; 1 SRS; 1 STA-R; 1 WAI | The therapist does not understand me.  He does not realize how strongly I feel about some of the things we discuss |
| Emotional experiencing | 12 | 2 CALPAS; 3 Menninger; 2 PSR; 1 RRI-C; 4 VPPS | The patient has a marked ability to experience and express clearly a broad range of intense emotions in the therapy session itself, including those specific to the therapist.  The patient shows a severly narrowed range of affective expressions |
| Idealisation/devaluation vs. realistic perception of therapist | 12 | 1 IRS; 2 Menninger; 1 PRQ; 2 PSR; 3 RRI-C; 2 RRI-T; 1 RRQ | Vacillates between idealising and devaluing the therapist.  I was able to separate out my realistic perceptions of my therapist from my unrealistic perceptions. |
| General affection/sympathy and acceptance towards therapist | 11 | 1 IRS; 1 ARM; 1 HAQ-2; 1 PRQ; 1 PSR; 3 RRI-T; 1 STAR-E; 1 TBS; 1 WAI-T | My client genuinely expresses his/her positive feelings toward me. |
| General aversion towards therapist | 7 | 1 ITASr-SF; 1 PRQ; 1 PSR; 2 RRI-T; 1 STAR-E; 1 STAR-D | I feel that my patient rejects me as a clinician. |
| Avoidance of relationship | 7 | 7 PRQ | Tries hard not to be, or feel, needy or dependent in therapy.  Seems to maintain distance from the therapist; tries to keep the therapist at arm’s length. |
| Friendliness, respect | 6 | 2 ARM; 1 RRI-T; 1 TBS; 1 WAI; 1 WAI-T | My client has respect for me as a person. |
| Being sexually attracted towards therapist | 6 | 6 PRQ | Is sexually attracted to the therapist |
| Entitlement | 6 | 5 PRQ; 1 VPPS | Requires or demands excessive contact, love, etc., from the therapist |
| Manipulative, controlling behavior | 6 | 1 3RS; 4 PRQ; 1 VPPS | Is manipulative |
| No gain of new insights | 5 | 1 ABAQ; 1 CALPAS; 1 HAQ-2; 1 PSR; 1 WAI-T | Did you have the impression that you were unable to deepen your understanding of what is bothering you? |
| Difficulties in relationships | 2 | 1 PRQ; 1 RRI-T | My client distorts the therapy relationship. |
| Other | 15 | 1 IRS; 5 PRQ; 1 RRI-C; 3 TBS; 5 VPPS |  |
| **Therapist terms** |  |  |  |
| Doesn’t consider patient’s needs/ abilities/ wishes | 49 | 4 AIA; 3 IRS; 4 ANS; 6 ARM; 6 CALPAS; 1 CEI; 1 CRF; 2 HAcs; 1 HAQ-2; 1 KAS-R; 2 Menninger; 9 RI; 1 RRI-C; 2 SRS; 1 STAR-E; 1 STAR-D; 1 STA-R; 1 VPPS; 1 WAI | My provider does not allow me to state my opinion.  He says more about himself than I am really interested to hear.  My therapist follows his/her own plans, ignoring my views of how to proceed. |
| Consideration of the patient’s needs/ abilities/ wishes | 48 | 1 3RS; 13 AiA; 1 IRS; 1 Brief-CALPAS; 5 CALPAS; 2 CRF; 1 HAS; 5 KAS; 2 KAS-R; 1 RI; 1 VTAS-S-R; 3 SRS; 2 STAR-E; 2 STAR-D; 1 TBS; 1 VPPS; 3 WAI; 2 WAI-T | My therapist checked with me to ensure that we were talking about the right topics for me to meet my goals.  My therapist checked with me to be sure that the way we addressed my concerns made sense to me. |
| Unconditional valuing of the patient/appreciation/respect | 39 | 1 IRS; 1 ANS; 2 ARM; 2 CALPAS; 1 CEI; 2 CRF; 2 HAcs; 2 HAr; 1 KAS-R; 1 Menninger; 10 RI; 2 RRQ; 1 SRS; 1 STAR-E; 1 STAR-D; 2 VPPS; 4 WAI; 3 WAI-T | The patient feels that the therapist respects and values him. |
| Stable support, | 38 | 1 3RS; 2 4PAS; 3 IRS; 2 ARM; 3 CALPAS; 2 CEI; 4 CRF; 4 HAcs; 3 HAQ-1; 1 HAQ-2; 2 HAr; 1 HAS; 1 ITASr-SF; 1 KAS; 1 KAS-R; 2 Menninger; 1 PRQ; 1 VTAS-S-R; 1 STAR-E; 1 STAR-D; 1 VPPS | I believe my doctor is helping me.  Therapist demonstrates commitment to help and confidence in treatment.  I feel I can count on my doctor. |
| Understanding, empathizing/empathy | 37 | 1 4PAS; 1 AiA; 2 IRS; 1 AROS; 1 Brief-CALPAS; 3 CALPAS; 2 HAcs; 1 HAQ-1; 1 HAQ-2; 1 HAr; 1 HAS; 1 Menninger; 9 RI; 1 RRI-C; 1 VTAS-S-R; 1 SRS; 1 STAR-E; 2 STAR-D; 1 TBS; 2 VPPS; 1 WAI; 1 WAI-T | He tries to see things through my eyes.  My therapist’s perceptions of me were accurate. |
| Openness, authenticity, honesty | 32 | 1 3RS; 1 IRS; 4 CRF; 1 HAr; 1 RDFS; 11 RI; 1 RRI-C; 4 RRI-T; 2 STAR-E; 2 STAR-D; 2 VPPS | He is willing to tell me his own thoughts and feelings when he is sure that I really want to know them. |
| Fosters new perspectives and dealings | 31 | 3 3RS; 1 IRS; 4 CALPAS; 3 CEI; 1 CRF; 1 HAcs; 1 HAr; 1 STA-R; 16 VPPS | The counselor’s comments helped me to see more clearly what I need to do to gain my objectives in life. |
| Plays a role, unknowable | 30 | 1 IRS; 4 CRF; 10 RI; 3 RRI-C; 3 RRI-T; 1 STAR-E; 1 STA-R; 1 VPPS; 1 WAI; 1 WAI-T | He adopts a professional role that makes it hard for me to know what he is like as a person.  He is uncomfortable when I ask him something about himself. |
| Fosters secure environment, trust | 28 | 1 ABAQ; 1 AiA; 2 IRS; 1 ANS; 6 ARM; 1 AROS; 1 CALPAS; 3 CRF; 2 Menninger; 1 PRQ; 1 RI; 2 RRI-C; 1 RRI-T; 2 RRQ; 3 VPPS | My therapist asked me how he/she could help me feel comfortable talking about my concerns. |
| Facilitates open communication | 26 | 3 3RS; 3 AiA; 6 ANS; 1 HAr; 2 KAS; 1 KAS-R; 1 Menninger; 1 PRQ; 5 RI; 1 RRI-C; 2 VPPS | Within the context of a rupture, the therapist invites the patient to discuss thoughts or feelings with respect to the therapist or some aspect of therapy. |
| Making patient weak | 24 | 4 IRS; 1 ANS; 1 AROS; 1 CEI; 1 CRF; 7 HAcs; 1 HAQ-2; 1 HAr; 1 HAS; 1 KAS-R; 2 Menninger; 1 STA-R; 2 VPPS | In our talks, the counselor acted as if he were better than I.  The therapist conveys that he does not accept the patient as someone who can use the basic tools of the treatment. |
| Unalertness/inattention, boredom | 24 | 1 ABAQ; 5 IRS; 2 ARM; 2 AROS; 1 CEI; 1 CRF; 3 HAcs; 1 KAS-R; 1 Menninger; 6 RI; 1 TBS | T seems bored  Inattentive and cold  He is curious about "the way I tick," but not really interested in me as a person. |
| Affection, friendliness, warm | 24 | 2 IRS; 2 ARM; 1 AROS; 3 CRF; 1 HAcs; 2 HAr; 1 PRQ; 5 RI; 1 STAR-E; 5 TBS; 1 VPPS | My therapist is warm and friendly with me.  He feels deep affection for me. |
| Doesn’t understand patient | 23 | 1 IRS; 2 ARM; 1 AROS; 2 HAcs; 2 ITASr-SF; 2 Menninger; 6 RI; 1 RRI-C; 1 SRS; 1 STAR-E; 1 STAR-D; 1 STA-R; 2 WAI | It is difficult for me to empathize with or relate to my patient’s problems.  I did not feel heard by the therapist, understood, and respected. |
| Aversion, coldness, distanced | 22 | 2 IRS; 1 AROS; 1 CEI; 4 CRF; 2 HAcs; 1 HAQ-2; 2 Menninger; 6 RI; 2 RRI-T; 1 SRS | I do not like my client as a person.  At times, the therapist seems distant. |
| Makings patient strong | 20 | 1 IRS; 4 ARM; 3 HAcs; 2 HAQ-1; 3 HAr; 2 KAS; 2 KAS-R; 3 VPPS | My provider encourages me to make decisions.  The therapist gives recognition where appropiate that the patient has in some way made some progess toward the patient’s goals. |
| Inappropriate explanations, intransparency | 20 | 1 ABAQ; 3 IRS; 4 ARM; 1 CALPAS; 1 RI; 1 STAR-E; 1 STAR-D; 1 VPPS; 4 WAI; 3 WAI-T | I wish _______ and I could clarify the purpose of our sessions. |
| Alertness/attention, interest | 17 | 1 AROS; 1 Brief-CALPAS; 1 CEI; 1 CRF; 1 HAcs; 2 RI; 1 SRS; 1 STAR-E; 1 STAR-D; 5 TBS; 2 VPPS | He is interested in me.  I listen to my patient.  Alert – unalert |
| Benevolence towards patient | 16 | 1 4PAS; 2 IRS; 1 AROS; 1 CALPAS; 2 CEI; 1 HAcs; 1 HAQ-1; 1 HAQ-2; 1 HAr; 1 Menninger; 1 RI; 1 WAI; 2 WAI-T | I am genuinely concerned for _______________'s welfare.  I feel the therapist wants me to achieve my goals. |
| Appropriate/comprehensive explanations/psychoeducation, transparency | 16 | 1 3RS; 2 4PAS; 1 IRS; 2 ARM; 1 CRF; 1 KAS; 2 KAS-R; 1 VPPS; 2 WAI; 3 WAI-T | It is easy to understand my provider’s instructions.  I feel my provider gives me enough information. |
| Conditional valuing of the patient, moodiness | 16 | 1 IRS; 1 HAQ-2; 2 Menninger; 1 PRQ; 9 RI; 1 VPPS; 1 WAI | Depending on his mood, he some-times responds to me with quite a lot more warmth and interest than he does at other times. |
| Hopefulness/ optimism/ confidence/ secureness | 15 | 2 ARM; 1 BQTA; 2 CALPAS; 1 HAcs; 2 HAQ-1; 1 HAr; 2 TBS; 1 VPPS; 3 WAI-T | I felt comitted to help the patient and had confidence in therapy.  The therapist conveys a sense of hopefulness that treatment goals can be achieved. |
| Unbalanced, uneasy, insecure, inferior | 15 | 3 IRS; 2 ARM; 3 CEI; 1 CRF; 2 RI; 1 STAR-E; 2 STAR-D; 1 WAI-T | The counselor acted uncertain of himself.  The counselor seemed restless while talking to me. |
| Unreliableness, indifference | 14 | 3 IRS; 1 AROS; 1 CALPAS; 1 CEI; 2 CRF; 2 HAcs; 2 Menninger; 2 RI | He is indifferent to me.  Reliable-unreliable |
| Balanced, at ease, self-secure, stable, relaxed | 14 | 3 IRS; 2 ARM; 3 CEI; 1 CRF; 2 RI; 1 STAR-E; 1 TBS; 1 VPPS | T is confident.  He is secure and comfortable in our relationship.  In opening our conversations, the counselor seemed relaxed and at ease. |
| Other traits or mood | 13 | 3 IRS; 7 CRF; 2 TBS; 1 VPPS | attractive – unattractive  Casual – formal  Cheerful |
| Competence, knowledge, experience | 12 | 2 ARM; 1 CALPAS; 6 CRF; 1 HAQ-2; 2 Menninger | Expert-inexpert |
| Doesn’t facilitate open communication, tabus | 11 | 1 AiA; 1 KAS-R; 7 RI; 1 VPPS; 1 WAI | My therapist avoided talking about what is going on in the room between us. |
| Incompetence, lack of knowledge and experience | 9 | 2 IRS; 1 CEI; 5 CRF; 1 RI | Experienced-inexperienced |
| Hopelessness | 6 | 1 AROS; 2 HAcs; 3 WAI-T | The therapist conveys a sense of lack of hopefulness that the patient’s goals can be achieved. |
| Doesn’t foster new perspective/dealings,hinders therapy success | 5 | 1 HAQ-2; 1 Menninger; 1 STA-R; 1 VPPS; 1 WAI | The therapist relates to me in ways that slow up the progress of the therapy. |
| Other | 5 | 1 ABAQ; 2 HAcs; 1 HAr; 1 RI |  |

**Notes.** The reported frequencies are based on the first rater’s results.
3RS = Rupture Resolution Rating System; 4PAS = 4-Point-Alliance-Scale; ABAQ = Attachment Based Alliance Questionnaire; AiA = Alliance in Action; ANS = Alliance Negotiation Scale; ARM = Agnew-Davies Relationship Measure; AROS = Alliance and Rupture Observation Scale; BQTA = Brief Questionnaire on Therapeutic Alliance; Brief-CALPAS = 5-Item California Psychotherapy Alliance Scales; CALPAS = California Psychotherapy Alliance Scales; CEI = Counselor Evaluation Inventory; CRF = Counselor Rating Form; HAcs = Helping Alliance Counting Signs; HAQ-1 = Helping Alliance Questionnaire 1; HAQ-2 = Helping Alliance Questionnaire 2; HAr = Helping Alliance Rating Method; HAS = Helping Alliance Scale; IRS = Interview Rating Scale; ITASr-SF = Individual Therapy Alliance Scale revised – Short Form; KAS = Kim Alliance Scale; KAS-R = Kim Alliance Scale – Revised; Menninger = Menninger Alliance Scales; PRQ = Psychotherapy Relationship Questionnaire; PSR = Psychotherapy Status Report; RDFS = Relational Depth Frequency Scale; RI = Relationship Inventory; RRI-C = Real Relationship Inventory – Client Version; RRI-T = Real Relationship Inventory - -Therapist Version; RRQ = Real Relationship Questionnaire; SRS = Session Rating Scale; STAR-D = Scale to Asses Therapeutic Relationship – German version; STAR-E = Scale to Asses Therapeutic Relationship – English version; STA-R = Skala Therapeutische Allianz – Revised; TA = Therapeutic Alliance; TBS = Therapeutic Bond Scales; VPPS = Vanderbilt Psychotherapy Process Scale; VTAS-S-R = Vanderbilt Therapeutic Alliance Scale – Short Form – Revised; WAI = Working Alliance Inventory; WAI-T = Working Alliance Inventory – Therapist Version.

**PRISMA Checklist**

| **Section and Topic** | **Item #** | **Checklist item** | | **Location where item is reported** | |
| --- | --- | --- | --- | --- | --- |
| **TITLE** | | |  | | |
| Title | 1 | Identify the report as a systematic review. | | title | |
| **ABSTRACT** | | |  | | |
| Abstract | 2 | See the PRISMA 2020 for Abstracts checklist. | | abstract | |
| **INTRODUCTION** | | | | |  |
| Rationale | 3 | Describe the rationale for the review in the context of existing knowledge. | | 1 | |
| Objectives | 4 | Provide an explicit statement of the objective(s) or question(s) the review addresses. | | 1 | |
| **METHODS** | | | | |  |
| Eligibility criteria | 5 | Specify the inclusion and exclusion criteria for the review and how studies were grouped for the syntheses. | | 2, 2.1.1 | |
| Information sources | 6 | Specify all databases, registers, websites, organisations, reference lists and other sources searched or consulted to identify studies. Specify the date when each source was last searched or consulted. | | 2.1.1.2, 2.1.2 | |
| Search strategy | 7 | Present the full search strategies for all databases, registers and websites, including any filters and limits used. | | 2.1.1.2, 2.1.2 | |
| Selection process | 8 | Specify the methods used to decide whether a study met the inclusion criteria of the review, including how many reviewers screened each record and each report retrieved, whether they worked independently, and if applicable, details of automation tools used in the process. | | 2.1.1.2, 2.1.2 | |
| Data collection process | 9 | Specify the methods used to collect data from reports, including how many reviewers collected data from each report, whether they worked independently, any processes for obtaining or confirming data from study investigators, and if applicable, details of automation tools used in the process. | | 2.1.1.2, 2.1.2 | |
| Data items | 10a | List and define all outcomes for which data were sought. Specify whether all results that were compatible with each outcome domain in each study were sought (e.g. for all measures, time points, analyses), and if not, the methods used to decide which results to collect. | | 2.1.1 | |
|  | 10b | List and define all other variables for which data were sought (e.g. participant and intervention characteristics, funding sources). Describe any assumptions made about any missing or unclear information. | | 2.1.1 | |
| Study risk of bias assessment | 11 | Specify the methods used to assess risk of bias in the included studies, including details of the tool(s) used, how many reviewers assessed each study and whether they worked independently, and if applicable, details of automation tools used in the process. | | NA | |
| Effect measures | 12 | Specify for each outcome the effect measure(s) (e.g. risk ratio, mean difference) used in the synthesis or presentation of results. | | Table 1 | |
| Synthesis methods | 13a | Describe the processes used to decide which studies were eligible for each synthesis (e.g. tabulating the study intervention characteristics and comparing against the planned groups for each synthesis (item #5)). | | 2.1.1.2, 2.1.2 | |
|  | 13b | Describe any methods required to prepare the data for presentation or synthesis, such as handling of missing summary statistics, or data conversions. | | 2.1.1.2 | |
|  | 13c | Describe any methods used to tabulate or visually display results of individual studies and syntheses. | | 2.1.2; 3.1 | |
|  | 13d | Describe any methods used to synthesize results and provide a rationale for the choice(s). If meta-analysis was performed, describe the model(s), method(s) to identify the presence and extent of statistical heterogeneity, and software package(s) used. | | 2.1.2; 3.1 | |
|  | 13e | Describe any methods used to explore possible causes of heterogeneity among study results (e.g. subgroup analysis, meta-regression). | | NA | |
|  | 13f | Describe any sensitivity analyses conducted to assess robustness of the synthesized results. | | NA | |
| Reporting bias assessment | 14 | Describe any methods used to assess risk of bias due to missing results in a synthesis (arising from reporting biases). | | NA | |
| Certainty assessment | 15 | Describe any methods used to assess certainty (or confidence) in the body of evidence for an outcome. | | NA | |
| **RESULTS** | | | | |  |
| Study selection | 16a | Describe the results of the search and selection process, from the number of records identified in the search to the number of studies included in the review, ideally using a flow diagram. | | 2.1.2 | |
|  | 16b | Cite studies that might appear to meet the inclusion criteria, but which were excluded, and explain why they were excluded. | | 2.1.1.2 | |
| Study characteristics | 17 | Cite each included study and present its characteristics. | | 3.1 | |
| Risk of bias in studies | 18 | Present assessments of risk of bias for each included study. | | NA | |
| Results of individual studies | 19 | For all outcomes, present, for each study: (a) summary statistics for each group (where appropriate) and (b) an effect estimate and its precision (e.g. confidence/credible interval), ideally using structured tables or plots. | | 3.1 | |
| Results of syntheses | 20a | For each synthesis, briefly summarise the characteristics and risk of bias among contributing studies. | | NA | |
|  | 20b | Present results of all statistical syntheses conducted. If meta-analysis was done, present for each the summary estimate and its precision (e.g. confidence/credible interval) and measures of statistical heterogeneity. If comparing groups, describe the direction of the effect. | | 3 | |
|  | 20c | Present results of all investigations of possible causes of heterogeneity among study results. | | 3 | |
|  | 20d | Present results of all sensitivity analyses conducted to assess the robustness of the synthesized results. | | NA | |
| Reporting biases | 21 | Present assessments of risk of bias due to missing results (arising from reporting biases) for each synthesis assessed. | | NA | |
| Certainty of evidence | 22 | Present assessments of certainty (or confidence) in the body of evidence for each outcome assessed. | | NA | |
| **DISCUSSION** | | | | |  |
| Discussion | 23a | Provide a general interpretation of the results in the context of other evidence. | | 4 | |
|  | 23b | Discuss any limitations of the evidence included in the review. | | 4 | |
|  | 23c | Discuss any limitations of the review processes used. | | 5 | |
|  | 23d | Discuss implications of the results for practice, policy, and future research. | | 4, 6 | |
| **OTHER INFORMATION** | | | | |  |
| Registration and protocol | 24a | Provide registration information for the review, including register name and registration number, or state that the review was not registered. | | 2 | |
|  | 24b | Indicate where the review protocol can be accessed, or state that a protocol was not prepared. | | NA | |
|  | 24c | Describe and explain any amendments to information provided at registration or in the protocol. | | NA | |
| Support | 25 | Describe sources of financial or non-financial support for the review, and the role of the funders or sponsors in the review. | | 11 | |
| Competing interests | 26 | Declare any competing interests of review authors. | | 9 | |
| Availability of data, code and other materials | 27 | Report which of the following are publicly available and where they can be found: template data collection forms; data extracted from included studies; data used for all analyses; analytic code; any other materials used in the review. | | Data availability statement | |

**PRISMA Checklist for Abstracts**

| **Section and Topic** | **Item #** | **Checklist item** | **Reported (Yes/No)** | |
| --- | --- | --- | --- | --- |
| **TITLE** | | | |  |
| Title | 1 | Identify the report as a systematic review. | Yes | |
| **BACKGROUND** | | | |  |
| Objectives | 2 | Provide an explicit statement of the main objective(s) or question(s) the review addresses. | Yes | |
| **METHODS** | | | |  |
| Eligibility criteria | 3 | Specify the inclusion and exclusion criteria for the review. | Yes | |
| Information sources | 4 | Specify the information sources (e.g. databases, registers) used to identify studies and the date when each was last searched. | Yes | |
| Risk of bias | 5 | Specify the methods used to assess risk of bias in the included studies. | No | |
| Synthesis of results | 6 | Specify the methods used to present and synthesise results. | Yes | |
| **RESULTS** | | | |  |
| Included studies | 7 | Give the total number of included studies and participants and summarise relevant characteristics of studies. | Yes | |
| Synthesis of results | 8 | Present results for main outcomes, preferably indicating the number of included studies and participants for each. If meta-analysis was done, report the summary estimate and confidence/credible interval. If comparing groups, indicate the direction of the effect (i.e. which group is favoured). | Yes | |
| **DISCUSSION** | | | |  |
| Limitations of evidence | 9 | Provide a brief summary of the limitations of the evidence included in the review (e.g. study risk of bias, inconsistency and imprecision). | No | |
| Interpretation | 10 | Provide a general interpretation of the results and important implications. | Yes | |
| **OTHER** | | | |  |
| Funding | 11 | Specify the primary source of funding for the review. | No | |
| Registration | 12 | Provide the register name and registration number. | No | |
